# Manipulating crystallization dynamics through chelating molecules for bright perovskite emitters

**DOI:** 10.1038/s41467-021-25092-7

**Published:** 2021-08-10

**Authors:** Yatao Zou, Pengpeng Teng, Weidong Xu, Guanhaojie Zheng, Weihua Lin, Jun Yin, Libor Kobera, Sabina Abbrent, Xiangchun Li, Julian A. Steele, Eduardo Solano, Maarten B. J. Roeffaers, Jun Li, Lei Cai, Chaoyang Kuang, Ivan G. Scheblykin, Jiri Brus, Kaibo Zheng, Ying Yang, Omar F. Mohammed, Osman M. Bakr, Tönu Pullerits, Sai Bai, Baoquan Sun, Feng Gao

**Affiliations:** 1 Department of Physics, Chemistry and Biology (IFM), Linköping University, Linköping, Sweden; 2 Jiangsu Key Laboratory for Carbon-Based Functional Materials and Devices, Institute of Functional Nano and Soft Materials (FUNSOM), Joint International Research Laboratory of Carbon-Based Functional Materials and Devices, Soochow University, Suzhou, Jiangsu People’s Republic of China; 3 State Key Laboratory of Mechanics and Control of Mechanical Structures, Nanjing University of Aeronautics and Astronautics, Nanjing, China; 4 Chemical Physics and NanoLund, Lund University, Lund, Sweden; 5 Division of Physical Science and Engineering, King Abdullah University of Science and Technology, Thuwal, Kingdom of Saudi Arabia; 6 Institute of Macromolecular Chemistry of the Czech Academy of Sciences, Prague 6, Czech Republic; 7 Key Laboratory for Organic Electronics and Information Displays, Institute of Advanced Materials (IAM), Jiangsu National Synergetic Innovation Center for Advanced Materials (SICAM), Nanjing University of Posts & Telecommunications, Nanjing, China; 8 cMACS, Department of Microbial and Molecular Systems, KU Leuven, Leuven, Belgium; 9 NCD-SWEET beamline, ALBA Synchrotron Light Source, Barcelona, Spain; 10 Department of Chemistry, Technical University of Denmark, Kongens Lyngby, Denmark; 11 State Key Lab of Silicon Materials, Zhejiang University, Hangzhou, P. R. China

**Keywords:** Electronic devices, Nanoscale materials, Lasers, LEDs and light sources

## Abstract

Molecular additives are widely utilized to minimize non-radiative recombination in metal halide perovskite emitters due to their passivation effects from chemical bonds with ionic defects. However, a general and puzzling observation that can hardly be rationalized by passivation alone is that most of the molecular additives enabling high-efficiency perovskite light-emitting diodes (PeLEDs) are chelating (multidentate) molecules, while their respective monodentate counterparts receive limited attention. Here, we reveal the largely ignored yet critical role of the chelate effect on governing crystallization dynamics of perovskite emitters and mitigating trap-mediated non-radiative losses. Specifically, we discover that the chelate effect enhances lead-additive coordination affinity, enabling the formation of thermodynamically stable intermediate phases and inhibiting halide coordination-driven perovskite nucleation. The retarded perovskite nucleation and crystal growth are key to high crystal quality and thus efficient electroluminescence. Our work elucidates the full effects of molecular additives on PeLEDs by uncovering the chelate effect as an important feature within perovskite crystallization. As such, we open new prospects for the rationalized screening of highly effective molecular additives.

## Abstract (web summary)

Multidentate molecular additives are widely used to passivate perovskite, yet the role of chelate effect is still unclear. Here, the authors investigate a wide range of additives with different coordination number and functional moieties to establish correlation between coordination affinity and perovskite crystallisation dynamics.

## Introduction

Introducing molecular additives into perovskite precursors has become one of the most effective and prevailing strategies to improve the performance of metal halide perovskite light-emitting diodes (PeLEDs), and recently has boosted the external quantum efficiency (EQE) to high values above ~20%^1–7^. The performance enhancement results from suppressed nonradiative recombination and/or improved light-out coupling efficiency due to morphological variation^6,8,9^. The former is generally believed to be associated with defect passivation—surface dangling bonds in perovskites are said to be healed by additional coordination or ionic bonding with the additives^1,10,11^, leading to the elimination of trap states. Accordingly, a wide range of passivating molecular additives has been investigated for PeLEDs, especially those containing Lewis base moieties such as amino- and carboxyl-functionalized molecules^8,12–15^. In addition, the critical factors underlying passivation effectiveness have been well identified, including hardness/softness of functional moieties^16^, steric hindrance determined by molecular configurations^17,18^, as well as hydrogen bonding between passivating functional groups and organic cations^7,19^.

Despite these advances in understanding molecular passivation, a widely observed phenomenon yet to be rationalized by passivation alone is that almost all the effective additives for perovskite emitters are chelating molecules that contain more than one electron-rich functional moiety^6,7,20^. Conversely, mono-functionalized counterparts have received far less attention despite exhibiting similar molecular configurations and identical passivating groups. This contrast implies additional factors are at play (beyond the influence of passivation) that critically affect radiative recombination in the resulting halide perovskites.

Inspirations can be drawn from recent work on perovskite photovoltaics and point toward the possibility that molecular additives can modify crystallization dynamics^21,22^, resulting in discrepancies in defect densities and hence different radiative recombination processes. For instance, modulating crystallization dynamics with Lewis basic solvents, such as dimethyl sulfoxide (DMSO) and 1-methyl-2-pyrrolidinone (NMP), have been intensively investigated^21–23^. However, the chemical composition of perovskite emitters is remarkably different from that of perovskite photovoltaics due to the required enhancement of quantum and/or dielectric confinement^4,24–26^. Consequently, the crystallization control strategies developed in perovskite photovoltaics cannot be expected to be straightforwardly translated to PeLEDs. A thorough understanding of how the molecular additives influence perovskite crystallization in PeLEDs is thus required to better rationalize where the optoelectronic improvements originate.

Here, we reveal that the effects of prevailing molecular additives on manipulating the crystallization process of perovskite emitters are largely underestimated. By comparing two highly effective chelating molecules with their respective mono-functionalized counterparts, we find that chelation enhanced lead-additive binding affinity enables the formation of thermodynamically stable intermediates, leading to effective inhibition of perovskite nucleation and retarded crystal growth. The retarded crystallization process results in highly emissive perovskite thin films and high-efficiency near-infrared (NIR) PeLEDs with EQEs over 20%. We further generalize our conclusions by investigating a wide range of additives with different coordination numbers and functional moieties. Our results fill the knowledge gap between chelating molecular additives and bright perovskite emitters, through establishing the correlation between lead-additive coordination affinity and perovskite crystallization dynamics. As such, we provide a universal guideline for tuning the crystallization dynamics for high-performance PeLEDs by leveraging the chelate effect.

## Results

### PeLED device performance and film characterization

**Fig. 1:**
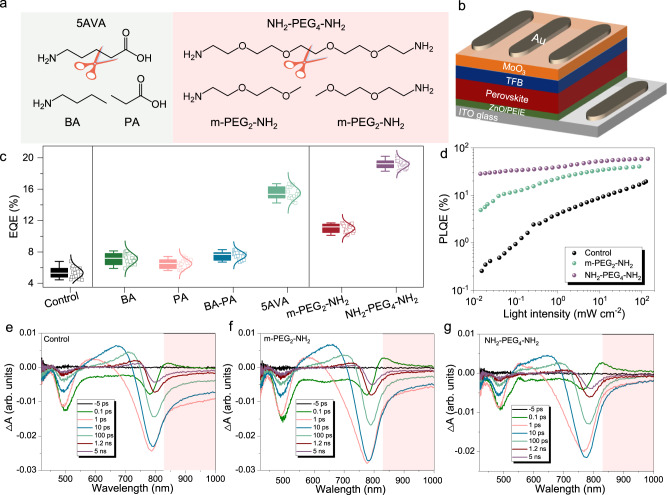
PeLED device architecture, performance, and perovskite film characteristics. **a** Molecular structures of the candidate chelating molecules (5AVA and NH_2_–PEG_4_–NH_2_), and their respective mono-functionalized counterparts (BA, PA, and m-PEG_2_–NH_2_). **b** A schematic of PeLED architecture. **c** EQE statistical distribution of 20 devices fabricated with different additives. **d**–**g** Photophysical studies for control, m-PEG_2_–NH_2_, and NH_2_–PEG_4_–NH_2_ perovskite thin films: fluence-dependent PLQE (**d**); TA spectra of control perovskite films (**e**), and the films with m-PEG_2_–NH_2_ (**f**) and NH_2_–PEG_4_–NH_2_ (**g**). The pink squares in (**e**–**g**) are to highlight the band tail absorption.

We compare the effects of two popular chelating additives (CAs) with their respective mono-functionalized additives (MFAs) on formamidinium lead tri-iodide (FAPbI_3_) emitters. The CAs we use are 5-aminovaleric acid (5AVA) and 3,6,9,12-tetraoxatetradecane-1,14-diamine (NH_2_–PEG_4_–NH_2_), which consist of commonly used amino- and/or carboxyl- groups; their respective mono-functionalized counterparts are *n*-butylamine (BA) and propionic acid (PA) for the former, and 2-(2-methoxyethoxy)ethanamine (m-PEG_2_–NH_2_) for the latter (Fig. 1a). Here, m-PEG_2_–NH_2_ is regarded as an MFA despite the electron-donating nature of the O atoms. This is based on the fact that the effects of oxygen atoms on crystallization are not as prominent as amino groups, as verified by comparing a counterpart with a longer polyethylene glycol (PEG) chain (2,5,8,11-tetraoxatridecan-13-amine, m-PEG_4_–NH_2_) (Supplementary Fig. 1 and Supplementary Note 1). All the additives were introduced into perovskite precursors at a mole ratio of PbI_2_:formamidinium iodide (FAI):additive = 1:2:*x* (*x* = 0–0.8). The use of excess organic components aims to passivate the vacancy defects, as widely demonstrated in PeLEDs^27,28^. As shown in the X-ray diffraction (XRD) patterns (Supplementary Fig. 2), the addition of additives does not change the three-dimensional structure of FAPbI_3_ at the optimal feed ratio for device fabrication. Besides, all the perovskite films show nano-island morphological features, although the addition of CA leads to smaller crystal sizes and denser surface coverage compared to the MFA-based counterparts (Supplementary Fig. 3). Our further investigations indicate that the morphological differences between MFA- and CA-films cannot be straightforwardly linked to the discrepancies in the device performance (Supplementary Fig. 4 and Supplementary Note 2).

We fabricate PeLEDs with additives and that without (named as the “control”) by subsequential deposition of zinc oxide (ZnO; 10 nm)/polyethyleneimine ethoxylated (PEIE)/FAPbI_3_/poly(9,9-dioctylfluorene-co-N-(4-(3-methylpropyl)) diphenylamine (TFB)/MoO_3_ (7 nm)/Au (80 nm) on indium tin oxide (ITO) substrates (see Fig. 1b and a cross-sectional image in Supplementary Fig. 5a). Devices based on the combined use of BA and PA at the mole ratio of 1:1 (named as the “BA-PA”) are also studied for comparison. The energy levels of the perovskite films are determined by ultraviolet photoelectron spectroscopy (UPS) spectra and a flat-band energy level diagram of devices (with control, m-PEG_2_-NH_2_ and NH_2_-PEG_4_-NH_2_ devices as the examples) are shown in Supplementary Fig. 5b–d and further discussed in Supplementary Note 3.

As the additives reduce the charge carrier injection to some extent due to their insulating nature (Supplementary Fig. 6), we carefully control their loading contents (see the details in Supplementary Table 1) to maximize the peak EQEs and simultaneously ensure a radiance as large as possible. We show a summary of average EQE values and detailed performance parameters with the optimized feed ratio in Fig. 1c and Supplementary Table 2, respectively, from which we find that CA addition results in considerable enhancement in terms of both radiance and EQE values compared to control and the respective MFA counterparts. Specifically, control devices show an average peak EQE of 5.3 ± 0.7%; 5AVA-devices give an average peak EQE of 15.5 ± 0.8% compared to 7.1 ± 0.7%, 6.5 ± 0.6%, and 7.6 ± 0.5% of BA, PA, and BA–PA mixture, respectively; NH_2_–PEG_4_–NH_2_devices have an average peak EQE of 19.2 ± 0.6% compared to 11.0 ± 0.5% of m-PEG_2_–NH_2_. The NH_2_–PEG_4_–NH_2_- and 5AVA-devices exhibit average radiance values of 360 ± 27 W sr^−1^ m^−2^ and 173 ± 20 W sr^−1^ m^−2^, which contrast sharply with the respective MFA devices (Supplementary Table 2). In addition, the champion NH_2_–PEG_4_–NH_2_- and 5AVA-devices reach high EQE values of 20.2% and 17.2%, respectively (see device characteristics in Supplementary Figs. 7 and 8). Intriguingly, BA–PA mixed precursors and double amounts of m-PEG_2_-NH_2_ do not give rise to comparable performance as the respective CA counterpart, suggesting the presence of additional underlying factors determining the device performance in addition to the amounts of Lewis base moieties. Moreover, CA devices show a prolonged operational lifetime with respect to the MFA counterparts measured under a constant current density of 20 mA cm^−2^ (Supplementary Fig. 9).

Given that m-PEG_2_–NH_2_ shares identical moieties with NH_2_–PEG_4_–NH_2_, we choose them as the main candidates for the rest of our investigations. We explore the photophysical properties of the perovskite films to further evaluate the radiative recombination and provide a rationale for the superior performance of CA devices. As shown in Fig. 1d, the NH_2_–PEG_4_–NH_2_ films display a much less pronounced dependence of photoluminescence quantum efficiencies (PLQEs) on excitation fluences with respect to its MFA counterparts and control films, giving the highest peak PLQE value up to 50%. More efficient radiative recombination in NH_2_–PEG_4_–NH_2_ films is also evident in PL mapping measurements^29,30^, from which we observe very homogeneous emission in space for the NH_2_–PEG_4_–NH_2_ film with time but distinct PL blinking in the control and m-PEG_2_–NH_2_ cases (Supplementary Fig. 10 and Supplementary Movies 1–3).

To further understand the discrepancy in the PL properties, we proceed to investigate the electronic states of the perovskite films using transient absorption (TA) measurements (Fig. 1e–g). In all the cases, we observe two photobleaching (PB) bands situated near 780 nm (PB1) and 490 nm (PB2), suggesting no significant alteration in their photophysical behavior with additives addition. The appearance of PB2 has been previously observed in bulk CH_3_NH_3_PbI_3_ perovskites, but the assignment remains unclear^31,32^. One previous report proposes that PB1 and PB2 arise from the transitions between two valence bands (VBs) to a common conduction band minimum (CBM)^31^. Notably, the control film shows a distinct band (>800 nm) in different delayed time scales, suggesting a relatively large number of sub-bandgap trap states^33^. The band tail absorption is reduced to some extent in m-PEG_2_–NH_2_ films but remarkably weakened in NH_2_–PEG_4_–NH_2_ incorporated films. Following the decreases in the band tail absorption, the PB1 is slightly blue-shifted compared to control and m-PEG_2_–NH_2_ cases, which can be assigned to the decoupling between PB1 and the less pronounced sub-bandgap trap states. Combining the results of PLQEs, PL mapping, and TA measurements, we conclude that trap-assisted nonradiative recombination is largely suppressed in NH_2_–PEG_4_–NH_2_ films, resulting in superior device performance.

### Understanding the effects of additives

The reduced trap-assisted nonradiative recombination could be attributed to efficient defect passivation, improved crystal quality, or a collective effect of them. By Fourier transform-infrared spectroscopy (FT-IR) measurements, we demonstrate that both m-PEG_2_–NH_2_ and NH_2_–PEG_4_–NH_2_ are retained in the perovskite films, as confirmed by the appearance of stretching vibration of ether bonds (C–O–C) at around 1110 cm^−1^ (Supplementary Fig. 11). The presence of additives provides opportunities for them to interact with defects chemically, contributing to the mitigated nonradiative recombination. It is further confirmed by the density of functional theory (DFT) calculations (Supplementary Figs. 12 and 13). Notably, our theoretical calculations suggest little differences in passivation effectiveness between these two additives, as further discussed in Supplementary Fig. 13 and Supplementary Note 4. This is also consistent with the fact that the passivation effects are usually determined by the type of functional groups and their chemical environment, both of which are identical in m-PEG_2_–NH_2_ and NH_2_–PEG_4_–NH_2_, as confirmed by calculation of the electronic property of the molecules (Supplementary Fig. 14). These results exclude the possibility that the significant difference in radiative recombination and device performance are the results of different passivation effectiveness between these two molecules.

**Fig. 2:**
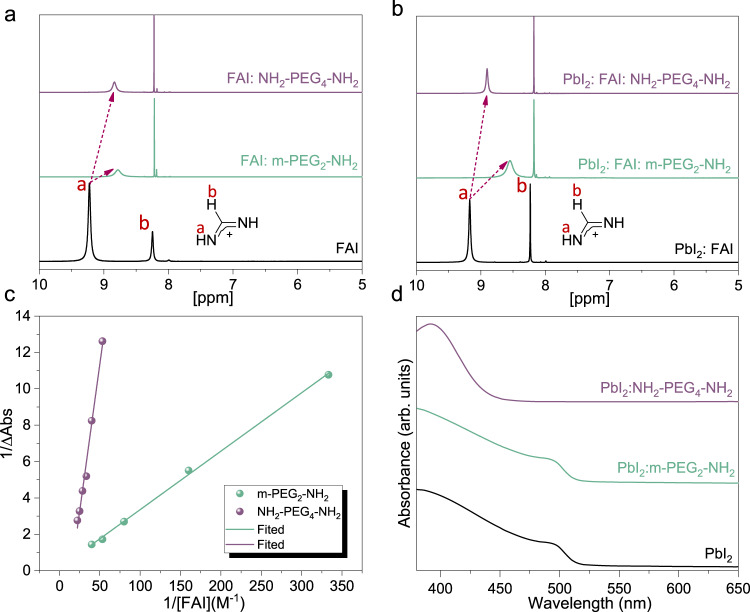
Interactions between the additives and perovskite precursors. **a**, **b**^1^H NMR of FAI, FAI:m-PEG_2_–NH_2_ (1:0.2) and FAI:NH_2_–PEG_4_–NH_2_ (1:0.1) (**a**) and PbI_2_:FAI (1:2), PbI_2_:FAI:m-PEG_2_–NH_2_ (1:2:0.4) and PbI_2_:FAI:NH_2_–PEG_4_–NH_2_ (1:2:0.2) (**b**). Peaks a and b are signals of H (**a**) and H (**b**) as labeled in the chemical structure of FA^+^ in the inset. **c** BH plots extracted from the traces of PbI_3_^−^ absorption (370 nm) with increasing FAI content in PbI_2_:NH_2_–PEG_4_–NH_2_ and PbI_2_:m-PEG_2_–NH_2_ parental solutions (Supplementary Fig. 16). **d** Ultraviolet–visible (UV–vis) absorption of neat PbI_2_ film, PbI_2_:m-PEG_2_–NH_2_ (1:0.4), and PbI_2_:NH_2_–PEG_4_–NH_2_ (1:0.2) films. All the stoichiometric ratios here are mole ratios.

With little difference in passivation effectiveness expected to occur between the use of CA and MFA additives, we focus our investigations on the effects of different molecules on the crystallization process. The first question that arises is whether these additives have different interaction strengths with the perovskite precursors; the interaction strength determines the intermediate phases and thus plays the key role in governing the crystallization process^21,34,35^. Amines can form hydrogen bonds with charged ammonium cations (FA^+^)^7^, which are characterized by ^1^H NMR. The number of Lewis base moieties is constant in all the samples, that is, at a mole ratio of 1:0.2 for FAI:m-PEG_2_–NH_2_ mixtures and 1:0.1 for FAI:NH_2_–PEG_4_–NH_2_ ones. The resonance signal of protonated ammonium peaks at 9.3 ppm in the pure FAI deuterated-DMF solution (Fig. 2a). In both cases with additive addition, the resonance signals of ammonium shift to 8.8 ppm, implying similar interaction strengths between both additives and FA^+^. These results are in line with the DFT calculations that the chemical environments of the amino groups and oxygen atoms in m-PEG_2_–NH_2_ and NH_2_–PEG_4_–NH_2_ are very close. In contrast, for the samples containing PbI_2_, the resonance of ammonium becomes less susceptible to NH_2_–PEG_4_–NH_2_ addition compared to m-PEG_2_–NH_2_, as adding the latter results in a much larger shift toward high field (Fig. 2b). These results imply that NH_2_–PEG_4_–NH_2_ has a stronger tendency than m-PEG_2_–NH_2_ to work with Pb^2+^ and thus leads to a weaker interaction with FA^+^ in the Pb^2+^-contained precursor solutions.

In this regard, we proceed to directly investigate the interaction between the additives and Pb^2+^. Adding NH_2_–PEG_4_–NH_2_ into PbI_2_ solution leads to the formation of white precipitation (Supplementary Fig. 15). This observation is consistent with the fact that amines are well-known building blocks for constructing metal amide complexes. Moreover, the yielded products will gradually diminish with FAI addition, indicating a competitive ligand exchange between the electron-rich moieties of additives and iodide anions from FAI.

We next quantified the interaction strength between Lewis base ligands and Pb^2+^ on the basis that iodide ions compete with Lewis base moieties to coordinate with Pb^2+^ ions^36^. We perform Benesi−Hildebrand (BH) analyses and determine the formation constant (*K*_f_) of iodoplumbate complex PbI_3_^−^ in the solution of interest^37^, by monitoring the Ultraviolet–visible (UV–vis) absorption evolution with increasing FAI content in the PbI_2_:additive parental solutions. We double the amounts of m-PEG_2_–NH_2_ with regards to NH_2_–PEG_4_–NH_2_ to keep Lewis base moieties constant in the solutions. By following the absorption spectra evolution with increasing FAI content, we observe gradually enhanced absorption intensity of PbI_3_^−^ in both cases (Supplementary Fig. 16). Notably, PbI_2_:NH_2_–PEG_4_–NH_2_ parental solution requires much higher amounts of FAI to achieve comparable absorbance to m-PEG_2_–NH_2_ ones. Figure 2c shows the BH plots, from which our calculations yield a larger *K*_f_ value (1/slope) of 31.1 M^−1^ for m-PEG_2_–NH_2_ solution compared to 3.1 M^−1^ for that with NH_2_–PEG_4_–NH_2_. These results confirm that the iodoplumbate complex is more difficult to form with the presence of NH_2_–PEG_4_–NH_2_, indicating a higher affinity between NH_2_–PEG_4_–NH_2_ and Pb^2+^. We attribute this to the chelate effect that the chelating ligands (e.g. NH_2_–PEG_4_–NH_2_) have a stronger affinity to the metal ions than a collection of their nonchelating or weak chelating counterparts (e.g. m-PEG_2_–NH_2_) despite identical functional groups, leading to kinetically more stable lead-additive coordination in the solutions.

Another important effect of the Pb^2+^–CA coordination with chelation enhanced affinity is the formation of thermodynamically stable lead complexes in the solid states. We show the UV–vis absorption spectra of PbI_2_: additive and neat PbI_2_ films in Fig. 2d and their XRD patterns in Supplementary Fig. 17. All these films were annealed at 100 °C for 10 min before measurements. Compared to the neat PbI_2_ film, the PbI_2_:NH_2_–PEG_4_–NH_2_ film turns to colorless (Supplementary Fig. 18), where the absorption onset shifts to a shorter wavelength of around ~450 nm (Fig. 2d). It also displays an amorphous feature with no discernible diffraction peaks, in sharp contrast to the PbI_2_ neat film which shows a typical crystalline (110) diffraction peak at 2*θ* = 12.6° (Supplementary Fig. 17). These results suggest that the addition of NH_2_–PEG_4_–NH_2_ reconstructs Pb^2+^ coordination to form complexes that are stable at high temperatures for perovskite crystallization. In contrast, the PbI_2_:m-PEG_2_–NH_2_ film shows no difference compared to the neat PbI_2_ film regarding both absorption and XRD patterns, indicating that m-PEG_2_–NH_2_ only has labile coordination to lead cations and that the formed complexes are readily decomposed at high temperatures needed for annealing perovskite films. We observe similar results in 5AVA and its MFA counterparts (BA, PA, and BA–PA combination). In particular, 5AVA:PbI_2_ films show identical blue-shifted absorption spectra and amorphous features as the NH_2_–PEG_4_–NH_2_ cases, while BA, PA, and BA–PA additions give no difference compared to neat PbI_2_ films (Supplementary Fig. 19).

**Fig. 3:**
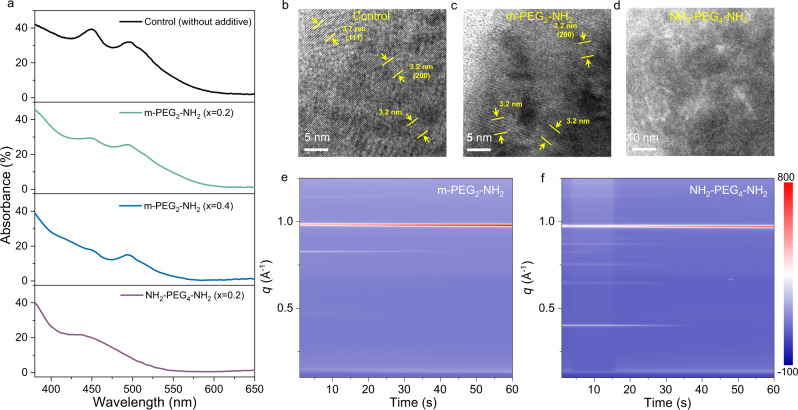
Analysis of perovskite nucleation and crystallization dynamics. **a** Ultraviolet–visible (UV–vis) absorption spectra of the control, PbI_2_:FAI:m-PEG_2_–NH_2_ (1:2:0.2 and 1:2:0.4) and PbI_2_:FAI:NH_2_–PEG_4_–NH_2_ (1:2:0.2) precursor films without annealing. **b**–**d** TEM images of the perovskite films before annealing. control (**b**); with m-PEG_2_–NH_2_ (**c**); with NH_2_–PEG_4_–NH_2_ (**d**). **e**, **f** Diffraction intensity map versus *q* with annealing time for perovskite films with a composition of PbI_2_:FAI:m-PEG_2_–NH_2_ = 1:2:0.4 (**e**) and PbI_2_:FAI:NH_2_–PEG_4_–NH_2_ = 1:2:0.2 (**f**). The films were deposited on ITO/ZnO/PEIE substrates. All the stoichiometric ratios here are mole ratios.

The formation of kinetically and thermodynamically more stable complexes, both attributed to the strong affinity of CAs to Pb^2+^, collectively affect the crystallization process of perovskites. Our characterizations show that the additives which exhibit a stronger affinity to lead cations are more effective in suppressing perovskite nucleation. The XRD patterns for the control and m-PEG_2_–NH_2_ (PbI_2_:FAI:m-PEG_2_–NH_2_ = 1:2:0.2) precursor films before annealing display the diffraction peaks from α-phase FAPbI_3_ (Supplementary Fig. 20a, b). We observe broad PL emission bands (Supplementary Fig. 21) peaking at shorter wavelengths compared to the typical 3D FAPbI_3_ in the control and m-PEG_2_–NH_2_-based precursor films, as well as two distinct excitonic absorption features peaking at ~450 and ~495 nm (Fig. 3a). Although the assignment of ~450 nm excitonic absorption is not clear, the other results clearly suggest the formation of α-FAPbI_3_ nanocrystals with size smaller than the Bohr radius. This can be directly observed by the transmittance electron microscopy (TEM) results (Fig. 3b, c), where (100) and (200) planes of FAPbI_3_ crystals are discernable in control- and MFA-precursor samples. It is worthy to mention that the perovskite nanocrystals form during the film formation process rather than in the precursor solutions, as no “Tyndall effect” is visible in the precursor solutions (Supplementary Fig. 22). In comparison, no signature of crystalline perovskites can be found from XRD patterns (Supplementary Fig. 20c) and TEM images (Fig. 3d) in the optimal NH_2_–PEG_4_–NH_2_ precursor films without annealing (PbI_2_:FAI:NH_2_–PEG_4_–NH_2_ = 1:2:0.2). Consistently, we do not observe any discernible excitonic absorption features from UV–vis absorption spectra (Fig. 3a). Due to the absence of perovskites in the precursor films, no PL can be detected. All these results indicate that the perovskite nucleation is completely suppressed at room temperature with the addition of NH_2_–PEG_4_–NH_2_ by forming kinetically and thermodynamically stable intermediate phases. Notably, although these results indicate that chelating ligands are effective in suppressing perovskite nucleation, we notice that suppression of nucleation at room temperature does not necessarily indicate a good film quality, as simply increasing the amount of nonchelating additive can also suppress perovskite nucleation yet without benefiting the film quality (Supplementary Fig. 23).

We further investigate the in situ crystallization process with different additives by monitoring the grazing-incidence wide-angle X-ray scattering (GIWAXS) pattern evolution during thermal annealing. Since the conversion from the intermediate phases to α-FAPbI_3_ perovskites is very fast at a high temperature (100 °C) for device fabrication, we slow down the process by continuously increasing the temperature from 88 to 100 °C in 60 s. We show the evolution of integrated diffraction intensities on annealing time in Fig. 3e, f. The m-PEG_2_–NH_2_ film (0.4 eq to lead content) initially shows two distinct scattering rings with *q* value of ~0.98 and ~0.83 Å^−1^, which correspond to α-FAPbI_3_ and non-perovskite δ-FAPbI_3_, respectively. The signatures of α-FAPbI_3_ and δ-FAPbI_3_ can be detected in the NH_2_–PEG_4_–NH_2_ sample at the initial stage as well, but with much weaker diffraction intensities, consistent with the XRD results that the perovskite nucleation can hardly occur at a low temperature (Supplementary Fig. 20c). In addition, five more weak diffraction signals at the *q* values of 0.40, 0.65, 0.74, 0.86, and 0.90 Å are visible, implying the coexistence of multiple crystalline intermediate phases during crystallization. Following the increase of annealing time, the diffraction signals from δ-FAPbI_3_ and intermediate phases gradually diminish until they eventually disappear, accompanied by the increases in α-FAPbI_3_ diffraction intensity. Notably, a much longer time is required for the saturation of (110) plane diffraction peak in the NH_2_–PEG_4_–NH_2_ film compared to m-PEG_2_–NH_2_. This is in line with the proposed competitive ligand exchange process that iodide anions need to replace Lewis base ligands to coordinate with lead cations, and thus higher energy is required for the formation of PbI_6_^4−^ octahedra and following perovskite nucleation^38^. In addition, we suggest that the ligand exchange process continually occurs during the crystallization process, leading to constrained crystal growth and hence small grain sizes (Supplementary Fig. 3). As such, we conclude that the chelating additives serve as more efficient crystallization inhibitors by leveraging their strong affinity to lead cations.

### Investigations of other Lewis base additives

In order to complete the whole picture and generalize our findings, we investigate a wide range of Lewis base additives. We start with DMSO, the most intensively used Lewis base additive in perovskite photovoltaics, where a colorless DMSO–PbI_2_–FAI intermediate phase is expected to form^21^. It is believed that the formation of the intermediate phase can retard perovskite crystallization and hence enable high film qualities. In contrast, we observe the appearance of red DMSO-precursor films in our case. The excitonic absorption features in the UV–vis absorption spectrum (Supplementary Fig. 24a) are identical to the films without any additives, indicating the formation of perovskite nanocrystals in the precursor films rather than DMSO–PbI_2_–FAI intermediate phases. The difference between the DMSO-precursor film in photovoltaics and that in LEDs in our case can be ascribed to the employment of excess FAI and relevant changes in coordination equilibrium in the films. In other words, as a result of the weak DMSO–PbI_2_ binding affinity, the DMSO–PbI_2_–FAI intermediate phase is not stable in a halide-rich environment that is typically used in PeLEDs. Consistently, we observe no positive effect on the device performance of PeLEDs with the DMSO addition (Supplementary Fig. 24).

**Fig. 4:**
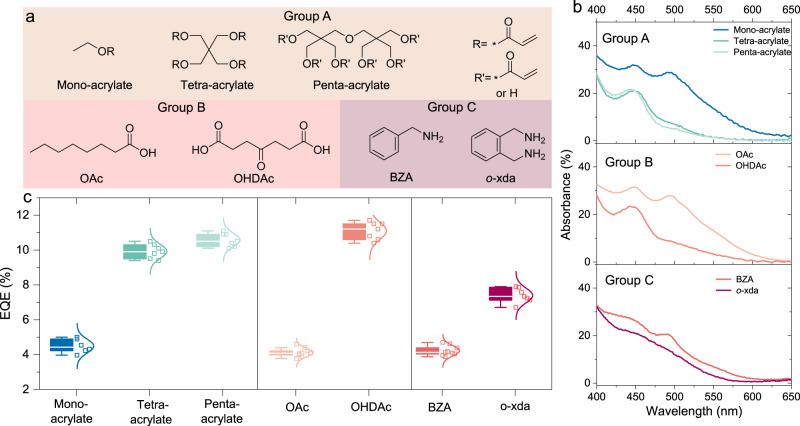
Dependence of device performance on the coordination number of additives and relevant affinity to lead cations. **a** Chemical structures of the alternative additives, including acrylates (ethyl acrylate (mono-acrylate), pentaerythritol tetraacrylate (tetraacrylate), and dipentaerythritol penta-acrylate (penta-acrylate), Group A), carboxylic acids (octane acid (OAc) and 4-oxoheptanedioic acid (OHDAc), Group B), and aromatic amines (benzylamine (BZA) and o-xylylenediamine (o-xda), Group C). **b** Ultraviolet–visible (UV–vis) absorption spectra for the precursor films (PbI_2_:FAI:additive = 1:2:*x*) with acrylates (*x* = 0.80, 0.20, 0.16 for mono-, tetra-, and penta-acrylates, respectively, top), carboxylic acids (*x* = 0.30, 0.10 for OAc and OHDAc, respectively, middle) and aromatic amines (*x* = 0.2 and 0.1 for BZA and o-xda, respectively, bottom). **c** A summary of average EQE values for the optimized devices based on various additives.

We proceed to investigate other Lewis base additives with different donating functional moieties and coordination numbers, including acrylates in Group A, carboxylic acids in Group B and aromatic amines in Group C (Fig. 4a). To estimate the dependence of lead-additive affinity on the coordination number of the additives in each group, we measure the UV–vis absorption spectra of the precursor films and keep the total amounts of functional moieties constant in each group by modifying the ratio of additives. With increasing coordination numbers, the absorption signatures from high-order iodoplumbate complex and/or crystalline perovskites gradually decrease (Fig. 4b), indicating enhanced lead-additive binding affinity. Due to the weak interaction between acrylates and lead cations, only those with high coordination numbers (e.g., tetra- and penta-acrylates) can influence the constituents of iodoplumbate complexes within the precursor films. We show the representative device characteristics in Supplementary Fig. 25. The averaged peak EQE values and all the detailed performance parameters are summarized in Fig. 4c and Supplementary Table 2, respectively. As expected, the device performance improves with increasing coordination numbers in each group. In addition, the dependence of device performance on the coordination numbers of additives is also visible in aromatic acids (Supplementary Fig. 26). All these results further confirm that increasing the lead-additive affinity by leveraging the chelate effect is a universal and effective strategy for preparing highly emissive perovskite thin films.

Having concluded the effectiveness and general applicability of chelation enhanced lead-additive affinity on improving film quality and device performance, the remaining question is if there is an upper limit to the affinity strength before film quality no longer benefits. We thus start with the bidentate chelating additive, hexamethylenediamine (HMDA), from which we enhance the affinity strength by moving to a multidentate chelating ligand, that is, triethylenetetramine (Trien) (see molecular structures in Supplementary Fig. 27). Trien is well-known for its superstrong affinity to metal ions and hence has been widely used to construct environmentally stable metal complexes^39^. Adding Trien into the perovskite precursor solutions leads to the immediate formation of white precipitation (Supplementary Fig. 28), indicating that the lead–Trien binding is too strong to be replaced by iodides. This result also indicates that an additive with too strong affinity may bring about impurities into the films. In contrast, HMDA addition has no negative impact on perovskite formation, and its binding affinity to lead cations is strong enough to suppress perovskite nucleation at room temperature by leveraging the chelate effect (Supplementary Fig. 29). The optimal HMDA-based devices give an average EQE of ~11% (Supplementary Fig. 30). These results indicate that an optimal range of coordination strength should be targeted, with a superstrong lead-additive affinity negatively impacting the perovskite formation.

## Discussion

**Fig. 5:**
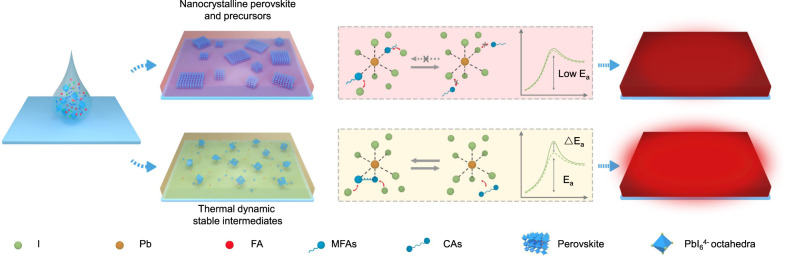
Schematic illustration of perovskite crystallization process with different lead-additive binding affinity. The additives with weak coordination affinities (e.g., MFAs) cause rapid perovskite nucleation at the initial stage of thermal annealing or even at room temperature. And those with strong affinity (e.g., CAs) bring about thermodynamically stable intermediates, resulting in highly emissive perovskites. The pink and yellow boxes illustrate the competitive ligand exchange between halides and additives with different binding affinity, as well as the respective evolution of thermal energy barrier (solid line) of perovskite crystallization compared to that without using additive (dash line). Here, *E*_a_ denotes the activation energy for crystallization, and Δ*E*_*a*_ is the enhanced energy caused by additive coordination.

Based on our collected findings, we present an overview of the correlation between lead-additive coordination affinity and crystallization dynamics in Fig. 5. In all scenarios, the additives compete with halide anions to coordinate with lead cations, resulting in an enhanced activation energy barrier (Δ*E*_*a*_) for perovskite nucleation due to the additional interactions involved in the formation of PbI_6_^4−^ octahedra. However, the halide-rich environment typically used for fabricating PeLEDs significantly hinders the coordination of additives and the relevant formation of intermediate phases. This becomes dominant for the case of using additives with weak coordination affinities, such as DMSO and the above-mentioned MFAs, resulting in little difference in the energy barrier for perovskite crystallization compared to those without using any additives. Consequently, perovskite nucleation and crystallization occur rapidly at the initial stage during annealing or even happen at room temperature, leading to a high density of defect sites and severe nonradiative recombination. In comparison, the strong lead-CA binding affinity enables effective ligand exchange, which takes over halide coordination. It thus gives rise to an enlarged energy barrier for perovskite nucleation by the formation of more thermodynamically and kinetically stable intermediate phases, slowing down the release of lead cations for perovskite crystallization during the annealing process. We also note that a multidentate additive with superstrong affinity may suppress the formation of perovskite crystals or bring about impurities that deteriorate the device performance. As such, for a specific precursor with a particular halide stoichiometry, tuning the lead-additive coordination affinity will be very useful for achieving optimal crystal quality. From a material design point of view, besides tuning coordination number of chelating molecular discussed here, other factors influencing the stability of metal complexes such as hard–soft nature of functional groups, macrocyclic effect, resonance effect, steric hindrance are all potential protocols for tuning the affinity and relevant crystallization dynamics of perovskites.

In summary, we have elucidated how molecular additives improve electroluminescence of perovskite emitters and developed a complete crystallization model which goes beyond defect passivation, by establishing the correlation between lead-additive coordination affinity and perovskite crystallization dynamics. We conclude that the additives with a strong binding affinity that leads to kinetically and thermodynamically stable intermediate phases are more effective in inhibiting perovskite nucleation and slowing down crystallization dynamics, which enables high crystal quality and thus efficient electroluminescence. This also indicates a different requirement in lead-additive binding affinity between perovskite emitters and photovoltaic absorbers due to the discrepancies in precursor stoichiometry. Our results rationalize the general and puzzling observation that chelating additives are much more widely used in state-of-the-art PeLEDs, and provide a universal guideline for tuning the crystallization dynamics for high-performance PeLEDs by leveraging the chelate effect.

## Methods

### Materials

Lead iodide (PbI_2_, anhydrous, 99.999%) was purchased from Alfa Aesar. Formamidinium iodide (FAI) was purchased from GreatCell Solar. Poly(9,9-dioctyl-fluorene-co-N-(4-butylphenyl)diphenylamine) (TFB) was purchased from American Dye Source. 2-(2-methoxyethoxy) ethanamine (m-PEG_2_–NH_2_), 2,5,8,11-tetraoxatridecan-13-amine (m-PEG_4_–NH_2_) and 3,6,9,12-tetraoxatetradecane-1,14-diamine (NH_2_–PEG_4_–NH_2_) were obtained from Biochempeg. Ethyl acrylate (mono-acrylate), pentaerythritol tetraacrylate (tetraacrylate), dipentaerythritol penta-acrylate (penta-acrylate), octane acid (OAc), 4-oxoheptanedioic acid (OHDAc), hexamethylenediaminedie (HMDA), triethylenetetramine (Trien), and all the other materials were purchased from Sigma-Aldrich. ZnO nanoparticles were synthesized as reported in the literature^40^.

### Preparation of perovskite solution

All the additives were introduced into perovskite precursors with a mole ratio of PbI_2_:formamidinium iodide (FAI):additives = 1:2:*x* (*x* = 0–0.8) in DMF to prepare the perovskite precursor solutions. The optimized feed ratio of additives for each type of device has been summarized in Supplementary Table 1.

### Device fabrication

Patterned ITO glass substrates were cleaned with deionized water and ethanol step by step, followed by UV–ozone treatment for 20 min before use. ZnO nanoparticles were spin-coated on the cleaned ITO substrates with 3000 r.p.m. for 30 s in ambient. Then, the ZnO-coated substrates were moved into an N_2_-filled glove box to deposit PEIE (1.1 mg mL^−1^ in isopropanol) at 3000 r.p.m. for 30 s, followed by annealing at 100 °C for 10 min. After cooling down, perovskite precursors were spin-coated at 3000 r.p.m. for 30 s and then annealed on a pre-heated hotplate at 100 °C for 10 min. TFB (12 mg mL^−1^ in chlorobenzene) was spin-coated on top of the perovskite films at 3000 r.p.m. for 30 s. After that, 7 nm MoO_3_ and 80 nm Au were deposited as the electrode in a thermal evaporator, the deposition rates are ~0.2 and ~0.5 Å s^−1^, respectively. For the electron-only devices, phenyl-C61-butyric acid methyl ester (PCBM, 30 mg mL^−1^ in chlorobenzene) was spin-coated on the top of the perovskite films at 3000 r.m.p for 30 s, followed by annealing at 70 °C for 10 min, followed by deposition of 100 nm Al. The active area of our PeLEDs is 7.25 mm^2^.

### Device characterization

The PeLEDs were measured at room temperature in an N_2_-filled glove box. A Keithley 2400 source meter incorporated with a QE Pro spectrometer (Ocean Optics) was used to measure the electrical output of PeLEDs. The applied voltage started from 0 V and increased with a step of 0.05 V, lasting for 300 ms at each voltage step for stabilization and measurements. The operational stability of the PeLEDs was measured under a constant current density of 20 mA cm^−2^ in the glove box.

### Grazing incident wide-angle X-ray scattering (GIWAXS)

GIWAXS data were obtained from NCD-SWEET beamline, ALBA Synchrotron, Spain. The energy of the X-ray beam was set to 12.95 keV using a Si (1 1 1) channel-cut monochromator and further collimated with an array of Be lenses. The incidence angle was 1° and the diffraction patterns were collected using a Rayonix^®^ LX255-HS area detector, which consists of a pixel array of 5760 × 1920 (V × H) with a pixel size of 88.54 × 88.54 μm^2^ for the pixel binning employed of 2 × 2. The scattering vector *q* was calibrated using Cr_2_O_3_ as standard, obtained using a sample to detector distance of 220 mm. All the samples were measured using a calibrated Linkam THMS600 heating stage adapted for grazing-incidence experiments under N_2_ atmosphere. The scan rate was one frame per two seconds.

### Transient absorption (TA)

The femtosecond transient absorption spectra were collected using a pump–probe configuration. A femtosecond oscillator (Mai Tai, Spectra Physics) is used as a seed laser and injected into a regenerative amplifier (Spitfire XP Pro, Spectra Physics) to generate a pulsed laser (800 nm, 80 fs pulse duration, 1 kHz repetition frequency). The second-harmonic generation (400 nm) from a BBO crystal was used as the pump. And the white light continuum (WLC) as the probe was produced by focusing the 800 nm fs pulse on a thin CaF_2_ plate. Polarization between the pump and probe was set to the magic angle (54.7°). During the experiments, gates on the pump intensity and probe spectra were set to avoid the influence of laser fluctuation on the measurements.

### Thin-film characterization

X-ray diffraction (XRD) patterns were conducted by an X-ray diffractometer (Panalytical X’Pert Pro) with CuK*α* radiation. The X-ray was generated on a Copper target, and the wavelength of the X-ray was 1.54 Å. Morphology images of the perovskite films were measured by an FEI (Quanta 200 FEG) scanning electron microscopy under a voltage of 5 kV. UV–vis absorbance spectra were collected from a PerkinElmer Lambda 900. All the precursor films for UV–vis measurements were encapsulated by glass slides and epoxy. Steady-state PL spectra were obtained with a 450-nm excitation laser and an Andor spectrometer (Shamrock sr-303i-B, coupled to a Newton EMCCD Si array detector). The fluence-dependent PLQEs were measured by a typical three-step technique with a combination of a 445-nm continuous-wave laser, spectrometer, and an integrating sphere.

The PL mapping measurements were conducted from a home-built wide-field epifluorescence microscope based on Olympus IX71 with a ×40 objective lens (Olympus LUCPlanFL, NA = 0.6). A 405-nm diode laser (CW) was used for excitation. The power density was approximately 0.25 W cm^−2^. The PL intensity as a function of time was recorded by a charge-coupled device camera (ProEM 512B, Princeton Instruments) with 100 ms of exposure time.

Fourier transform-infrared (FT-IR) spectra of perovskite films on ITO/ZnO/PEIE were conducted by a PIKE MIRacle Vertex 70 Spectrometer (Bruker) using a DLaTGS detector at room temperature. The spectrometer is equipped with a PIKE MIRacle^TM^ attenuated total reflectance (ATR) accessory as the sampling accessory. The measuring system was continuously purged with N_2_ before and during the measurements. The spectra were acquired at 2 cm^−1^ resolution with 30 scans over a wavenumber range between 4000 and 600 cm^−1^.

Transmittance electron microscopy (TEM) of precursor films was performed by a TECNAI G2 F20 transmission electron microscope with an accelerating voltage of 200 kV and a Gatan SC200 CCD camera. Ultraviolet photoelectron spectroscopy (UPS) of perovskite films were recorded by a multifunctional photoelectron spectrometer (PHI 5000 Versaprobe II) with a He I ultraviolet radiation source (21.2 eV).

### Nuclear magnetic resonance (NMR)

High-resolution ^1^H NMR data were recorded on a Bruker 600 MHz Avance III spectrometer at Larmor frequencies ν(^1^H) = 600.274 MHz. ^1^H NMR measurements single pulse experiments with 90° pulse of 18 μs, relaxation delay 10 s, 64 scans were applied. Dried D^7^-DMF (99.5%) was used as the solvent and was also used to calibrate isotropic chemical shifts. The temperature was kept constant at 298 ± 0.2 K with a BVT 3000 temperature unit during measurements. All samples were dissolved in dried DMF at room temperature in an inert (Ar) atmosphere. The concentration for Pb^2+^ and FAI is 0.12 and 0.24 M, respectively.

### UV–vis absorbance spectra for solution and Benesi−Hildebrand (BH) analyses

UV–vis absorbance spectra for solutions were collected by a PerkinElmer Lambda 35 at room temperature. Anhydrous DMF was used as the solvent. The concentration of Pb^2+^ for all the solutions is fixed at 0.25 mM. The mole ratio of PbI_2_:m-PEG_2_–NH_2_ and PbI_2_:NH_2_–PEG_4_–NH_2_ parent solution is 1:10 and 1:5, respectively. The formation equilibrium constant (K_f_) of PbI_3_^−^ was deduced by the Benesi−Hildebrand equation stated below:

1 $$\frac{1}{A-{A}_{0}}=\frac{1}{{K}_{f}\left({A}_{{\max }}-{A}_{0}\right)[{{{{{{\mathrm{FAI}}}}}}}]}+\frac{1}{{A}_{{\max }}-{A}_{0}}$$

Here, *A*_0_ is PbI_3_^−^ absorbance of the parent solution without FAI addition; *A* is the collected absorbance with FAI; *A*_max_ is the saturated absorbance with FAI addition; *K*_f_ is deduced from the slope of Benesi−Hildebrand plot.

### Density-functional theory (DFT) calculations

We performed the density-functional theory (DFT) calculations by using the projector-augmented wave (PAW) method as implemented in Vienna Ab initio Simulation Package (VASP)^41,42^. The generalized gradient approximation (GGA) with Perdew–Burke–Ernzerhof (PBE) method was used to optimize the crystal structures of cubic-phase FAPbI_3_ bulk, slabs, and interfaces. The wave functions were expanded on a plane-wave basis set up to a kinetic energy cutoff of 450 eV. The van der Waals functional (vdw) was also included in the structural optimizations and electronic calculations using the zero-damping DFT-D3 method of Grimme. Monkhorst–Pack-type K-meshes of 6 × 6 × 6 for the bulk FAPbI_3_, 4 × 4 × 1 for the FAPbI_3_ (110) slabs with different terminations, and 2 × 2 × 1 for the super-slabs and interfaces with m-PEG_2_–NH_2_ or m-PEG_4_–NH_2_ modification. All the structures were optimized until the forces on every single atom were less than 0.01 eV/Å. The binding energies were defined as *E*_B_ = *E*_interface_ – *E*_FAPbI3_ – *E*_ligand_, where *E*_interface_ is the total energy of the interface after m-PEG_2_–NH_2_ or m-PEG_4_–NH_2_ passivation, and *E*_FAPbI3_ and *E*_ligand_ represent the total energies of the individual parts. The molecular graphics viewer VESTA was used to plot the crystal structures.

The geometrical and electronic properties of additives were calculated by B3LYP (Becke Three Parameters Hybrid Functional with Lee–Yang–Perdew correlation functions) functional and 6–31G* basis set with Gaussian 09 program package. The electrostatic potential surfaces were visualized and drawn using Multiwfn and VMD software.

## Supplementary information

Supplementary Information

Description of Additional Supplementary Files

Supplementary Movie 1

Supplementary Movie 2

Supplementary Movie 3

## Data Availability

The data that support the plots within this paper and other findings of this study are available from the corresponding author upon reasonable request.
