# Supplementary material for: Manipulating crystallization dynamics through chelating molecules for bright perovskite emitters
Source: Nat Commun. 2021 Aug 10;12:4831. doi: 10.1038/s41467-021-25092-7 (PMC8355273; doi:10.1038/s41467-021-25092-7)
Supplement: Supplementary file 1 — Supplementary Information [file 41467_2021_25092_MOESM1_ESM.docx]

**Supplementary Information**

**Manipulating crystallization dynamics of metal halide perovskite emitters through chelation enhanced lead-additive affinity**

Yatao Zou^1,2,12^, Pengpeng Teng^1,3,12^, Weidong Xu^1^*, Guanhaojie Zheng^1^, Weihua Lin^4^, Jun Yin^5^, Libor Kobera^6^, Sabina Abbrent^6^, Xiangchun Li^7^, Julian A. Steele^8^, Eduardo Solano^9^, Maarten B. J. Roeffaers^8^, Jun Li^4^, Lei Cai^2^, Chaoyang Kuang^1^, Ivan Scheblykin^4^, Jiri Brus^6^, Kaibo Zheng^4,10^, Ying Yang^3^, Omar F. Mohammed^5^, Osman M. Bakr^5^, Tönu Pullerits^4^, Sai Bai^1,11^, Baoquan Sun^2^* and Feng Gao^1^*

^1^Department of Physics, Chemistry and Biology (IFM), Linköping University, Linköping, Sweden.

^2^Jiangsu Key Laboratory for Carbon-Based Functional Materials and Devices, Institute of Functional Nano and Soft Materials (FUNSOM), Joint International Research Laboratory of Carbon-Based Functional Materials and Devices, Soochow University, 199 Ren’ai Road, Suzhou, 215123, Jiangsu, People’s Republic of China.

^3^State Key Laboratory of Mechanics and Control of Mechanical Structures, Nanjing University of Aeronautics and Astronautics, Nanjing, China.

^4^Chemical Physics and NanoLund, Lund University, Lund, Sweden.

^5^Division of Physical Science and Engineering, King Abdullah University of Science and Technology. Thuwal 23955-6900, Kingdom of Saudi Arabia.

^6^Institute of Macromolecular Chemistry of the Czech Academy of Sciences, Heyrovskeho nam. 2, 162 06, Prague 6, Czech Republic.

^7^Key Laboratory for Organic Electronics and Information Displays, Institute of Advanced Materials (IAM), Jiangsu National Synergetic Innovation Center for Advanced Materials (SICAM), Nanjing University of Posts & Telecommunications, 9 Wenyuan Road, Nanjing 210023, China.

^8^cMACS, Department of Microbial and Molecular Systems, KU Leuven, 3001 Leuven, Belgium

^9^NCD-SWEET beamline, ALBA synchrotron light source, 08290, Cerdanyola del Vallès, Barcelona, Spain

^10^Department of Chemistry, Technical University of Denmark, DK-2800 Kongens Lyngby, Denmark.

^11^State Key Lab of Silicon Materials, Zhejiang University, Hangzhou 310027, P. R. China.

^12^These authors contributed equally: Y. Z. and P. T..

*Correspondence to W.X. (weidong.xu@liu.se); B.S. (bqsun@suda.edu.cn); F. G. (feng.gao@liu.se)

**Supplementary Figures**


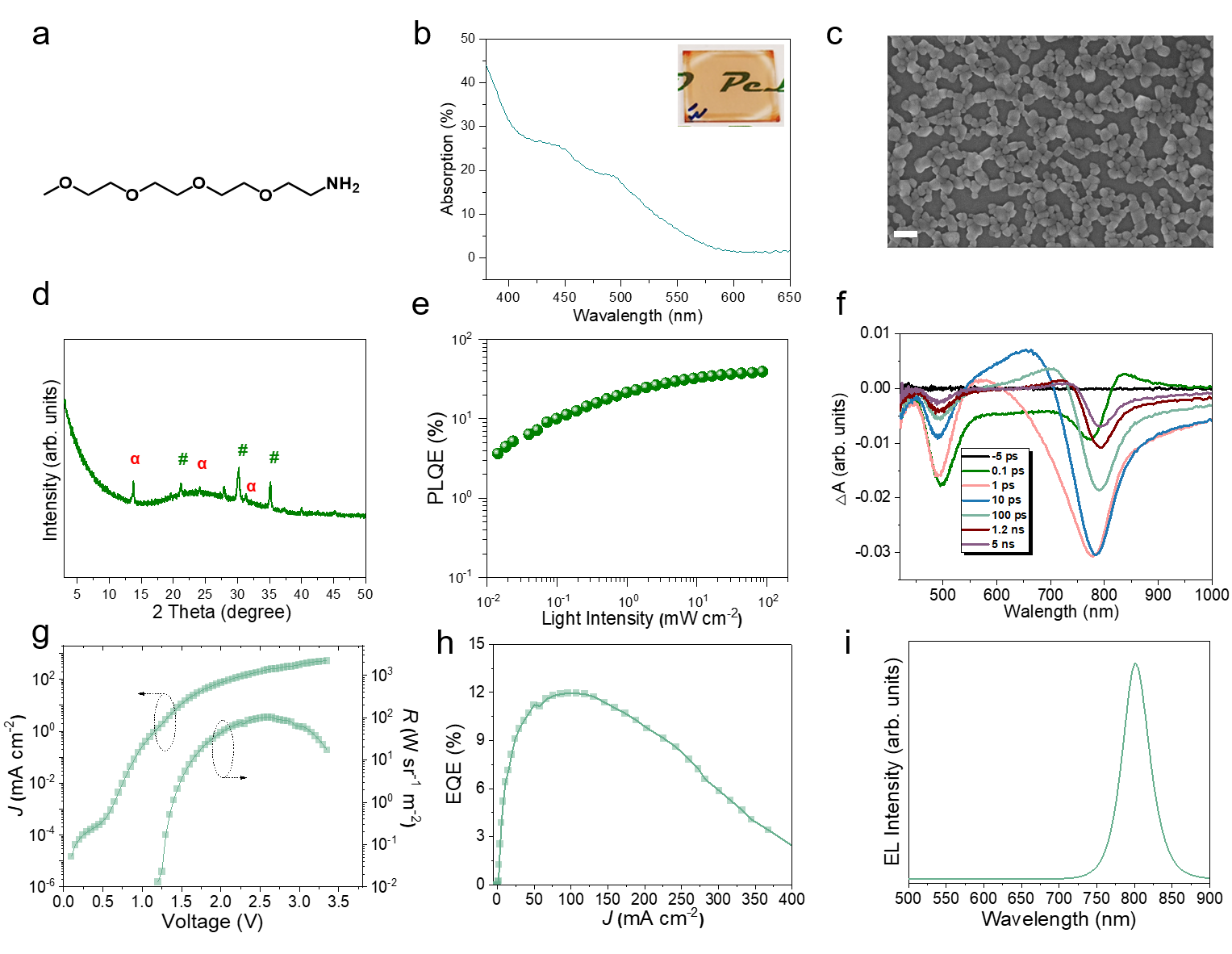


**Supplementary Figure 1 Characterizations of perovskite films and PeLEDs prepared with m-PEG_4_-NH_2_ as additive. a,** Molecular structure of m-PEG_4_-NH_2_. **b,** UV-vis absorption spectra of the perovskite precursor film before annealing. The inset shows the corresponding digital image of the perovskite precursor film. **c**, Top view SEM image. Scale bar: 200 nm. **d**, XRD patterns. Here, # donates the diffraction from ITO, and α donates the diffraction peaks from α phase of FAPbI_3_. **e**, Fluence-dependent PLQEs. **f**, Time-resolved transient absorption spectra. **g-i**, Characteristics of representative m-PEG_4_-NH_2_ based PeLED: Current density and radiance as a function of voltage (*J-V-R*) (**g**); EQE as a function of current density (*J*-EQE) (**h**); Normalized EL spectra (**i**). All the films and devices were prepared from precursors at an optimized stoichiometry as summarized in Supplementary Table 1.


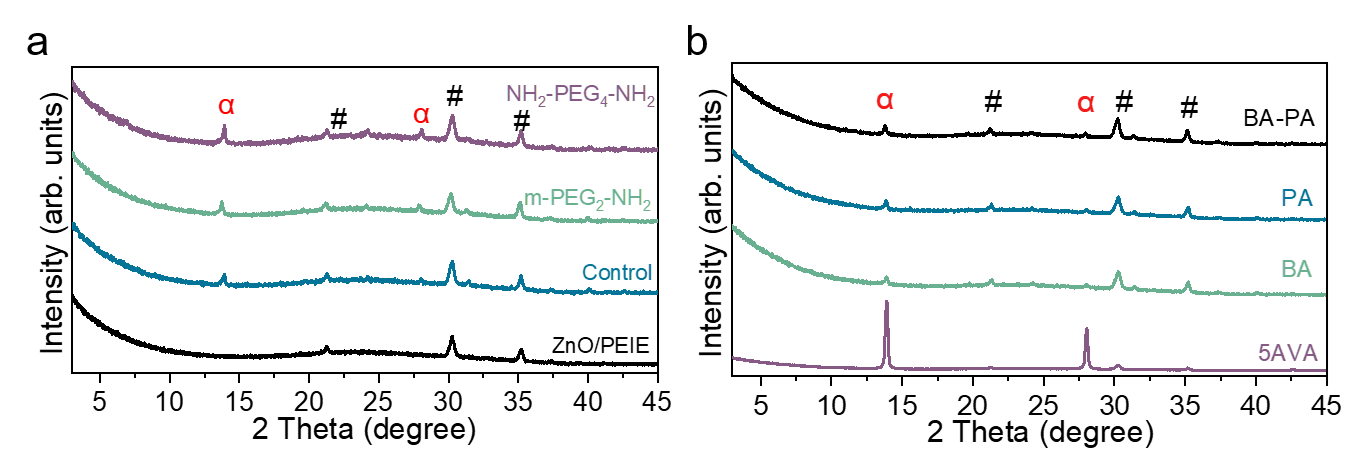


**Supplementary Figure 2** **XRD patterns of perovskite films prepared from chelating additives (NH_2_-PEG_4_-NH_2_, 5AVA) and their respective mono-functionalized additives (m-PEG_2_-NH_2_, BA, PA, and a combination of BA and PA)**. **a**, NH_2_-PEG_4_-NH_2_, m-PEG_2_-NH_2_, control films, and ITO/ZnO/PEIE substrate. **b**, 5AVA, BA, PA, and BA-PA mixed (1:1 in mole) films. Here, # donates the diffraction from ITO, α donates the diffraction peaks from α phase of FAPbI_3_. All the perovskite films were prepared from the respective optimal precursor stoichiometry as summarized in Supplementary Table 1.


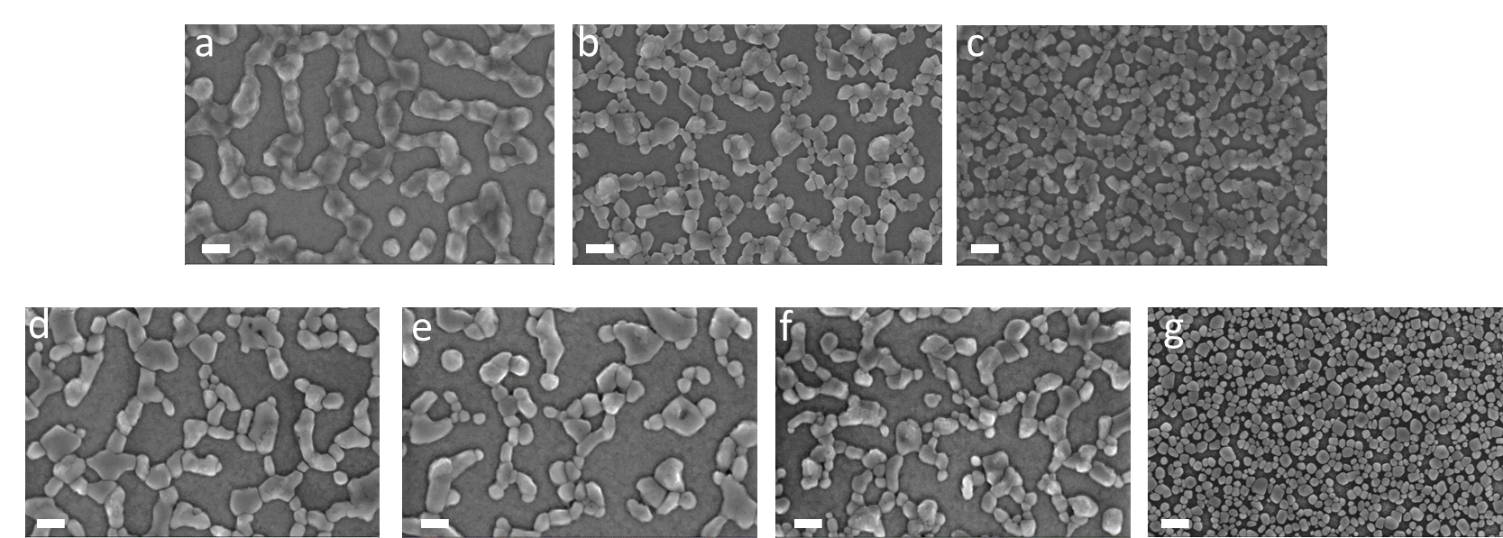


**Supplementary Figure 3 Top view SEM images of perovskite films prepared from various additives**. **a**, Control. **b**, m-PEG_2_-NH_2_. **c**, NH_2_-PEG_4_-NH_2_. **d**, BA. **e**, PA. **f**, BA-PA. **g**, 5AVA. Scale bars: 400 nm. All the samples were deposited on ITO/ZnO/PEIE substrates. All the perovskite films were prepared from the respective optimal precursor stoichiometry as summarized in Supplementary Table 1.


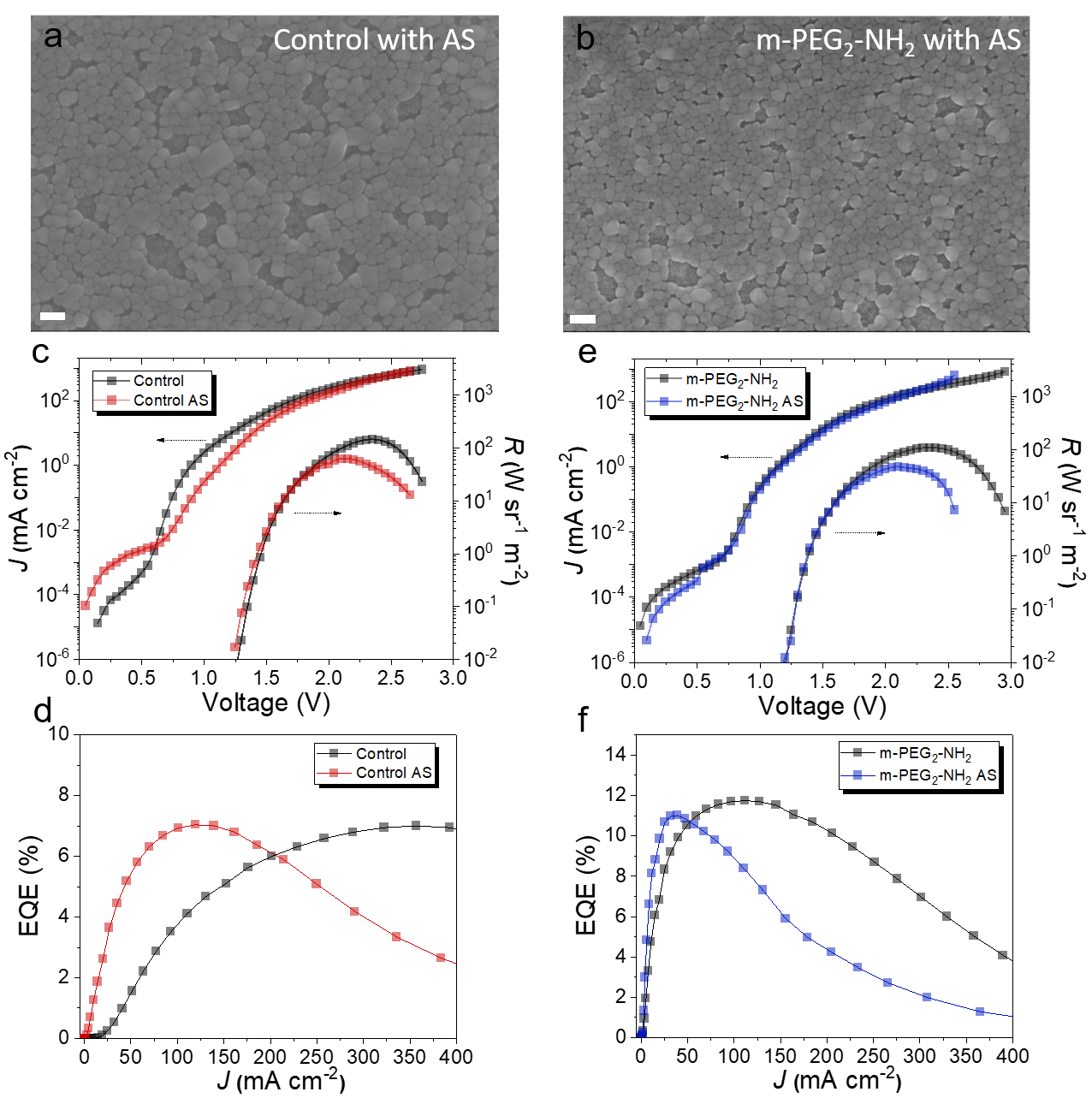


**Supplementary Figure 4 Understanding the perovskite film morphology on the device performance.** **a-b,** SEM images of perovskite films with anti-solvent (AS) treatment during the spin-coating process. Scale bars are 200 nm. Control (**a**); m-PEG_2_-NH_2_ (**b**). **c-f,** Characterizations of representative devices with AS treatment. *J-V-R* curves (**c** and **e**); *J*-EQE curves (**d** and **f**).


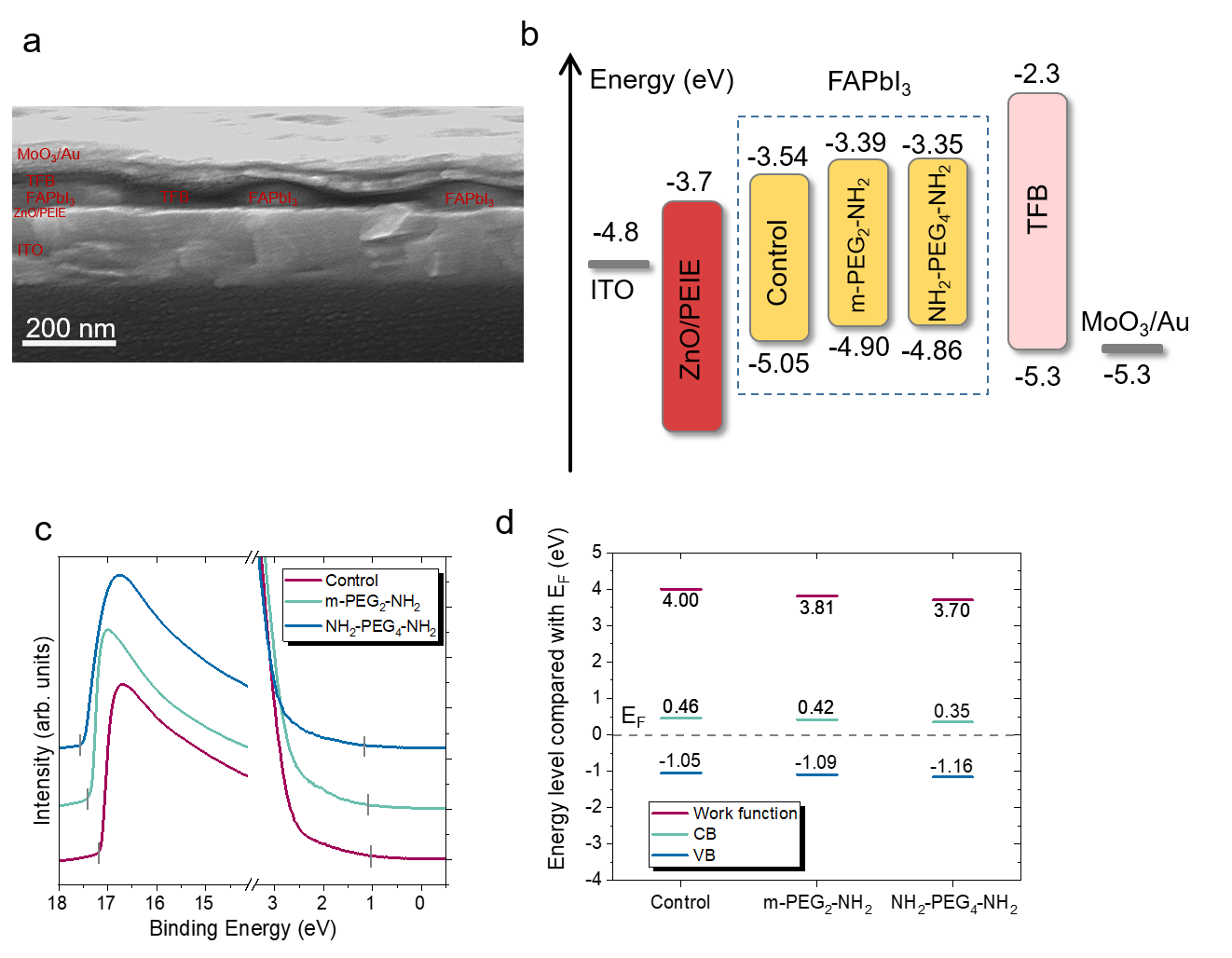


**Supplementary Figure 5 Architecture of PeLEDs**. **a**, A cross-sectional SEM image of an NH_2_-PEG_4_-NH_2_-device. **b**, Schematic illustration of flat-band energy level diagram of PeLEDs. The energy level of perovskite films is extracted from UPS spectra in Supplementary Fig. 5c. The energy level of ITO, ZnO/PEIE, TFB, and MoO_3_/Au are taken from reference^1^. **c**, UPS spectra (He I= 21.22 eV) of perovskite films with different additives (Control, m-PEG_2_-NH_2_, NH_2_-PEG_4_-NH_2_). **d**, Schematic energy diagram of the valence band (VB), conduction band (CB), and work function (WF) with respect to Fermi energy (E_F_). The CB levels are determined by the optical bandgap of our FAPbI_3_ films (~1.51 eV). All the films were prepared from precursors at an optimized stoichiometry as summarized in Supplementary Table 1.


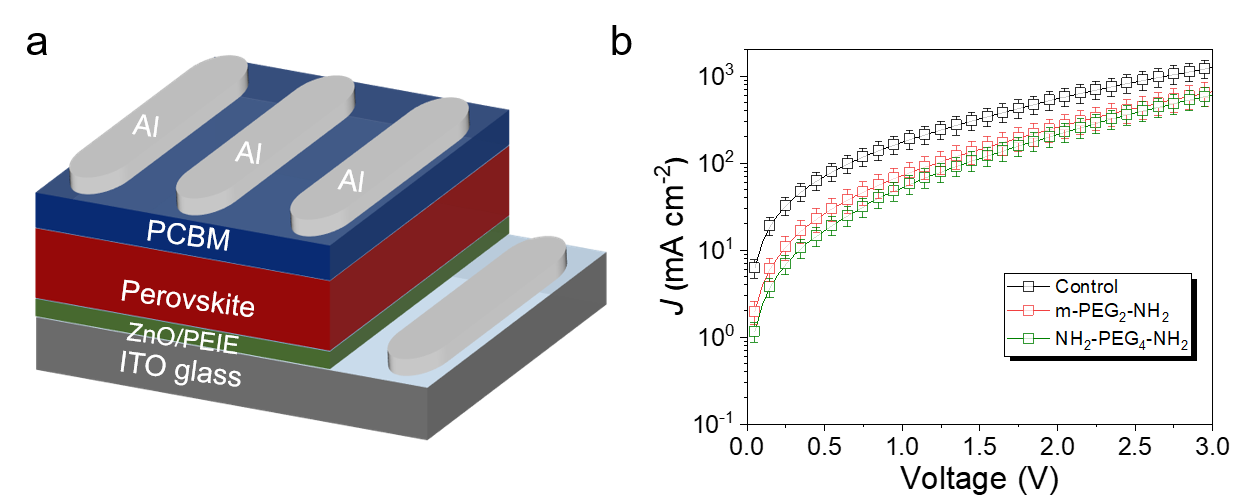


**Supplementary Figure 6. The effect of additive on charge carrier transport.** **a,** An illustration of electron-only devices with an architecture of ITO/ZnO/PEIE/perovskite/PCBM/Al. **b,** *J-V* curves for electron-only devices with control, m-PEG_2_-NH_2_, NH_2_-PEG_4_-NH_2_ -based perovskites. The error bars represent the standard deviation.


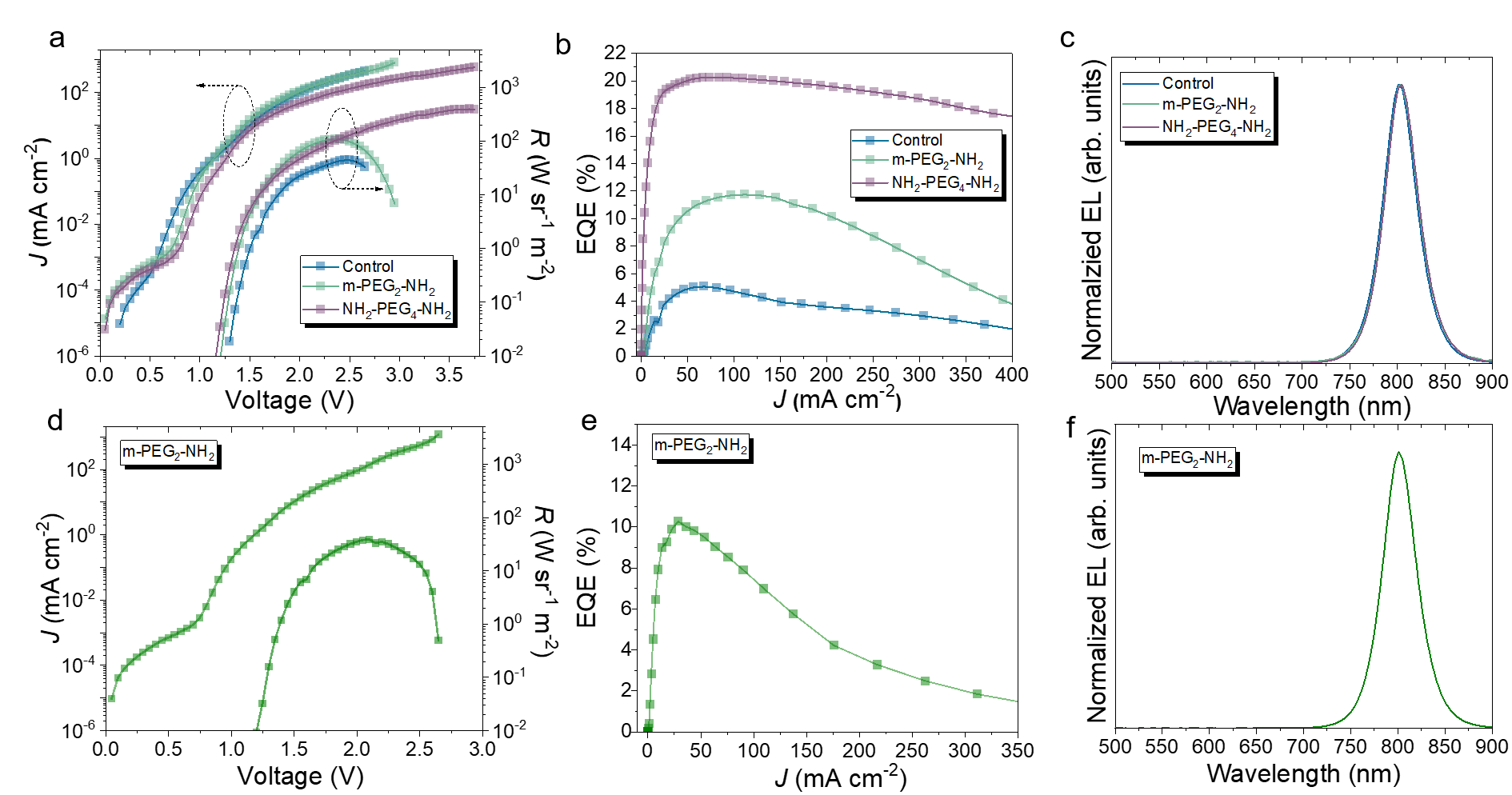


**Supplementary Figure 7 Characterizations of representative devices with different additives. a-c,** Optimal control, m-PEG_2_-NH_2_-_,_ and NH_2_-PEG_4_-NH_2_-based PeLEDs. The optimized feed ratio of PbI_2_: FAI: additive is 1: 2: 0.2 for both cases. **d-f,** PeLEDs prepared from precursors with m-PEG_2_-NH_2_, of which the feed ratio (0.4 equivalent of lead) is twice as much as the optimal NH_2_-PEG_4_-NH_2_ content (0.2 equivalent of lead). Device characteristics: *J-V-R* (**a**, **d**); *J*-EQE curves (**b**, **e**); Normalized EL spectra (**c**, **f**).


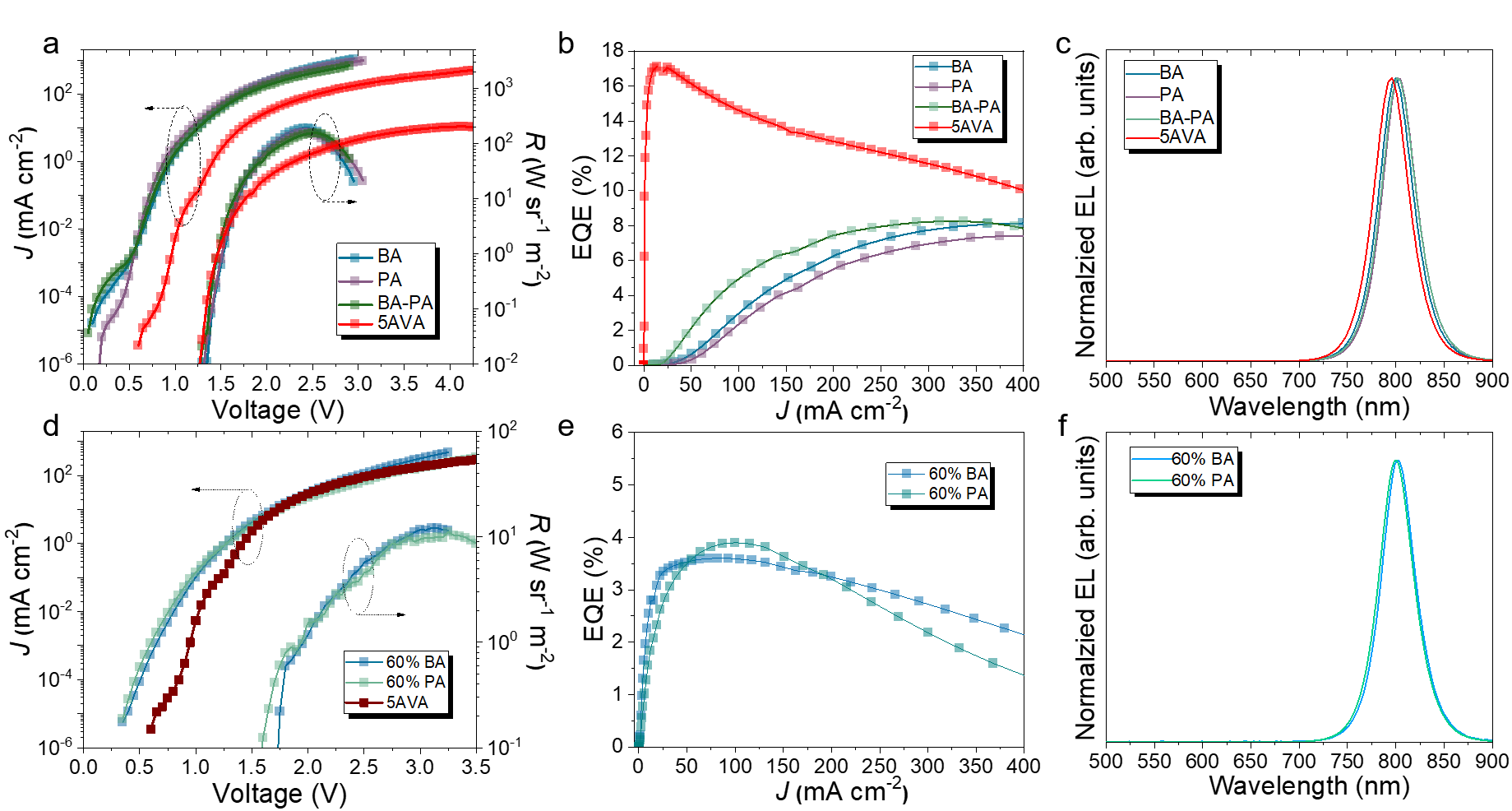


**Supplementary Figure 8 Characterizations of representative devices with different additives. a-c,** 5AVA, BA, PA, and BA-PA mixed (1:1 in mole) based PeLEDs. Here, the optimized ratio of PbI_2_: FAI: additive is 1: 2: 0.6 for 5AVA, 1: 2: 0.4 for BA, 1: 2: 0.1 for PA and 1: 2: 0.2 for BA-PA respectively. The optimized feed ratio is determined by considering both peak EQE values and maximum radiance. **d-f,** PeLEDs prepared from precursors with high ratio BA and PA, of which the feed ratio (0.6 equivalent of lead) is the same as the optimal 5AVA content (0.6 equivalent of lead). Device characteristics: *J-V-R* (**a**, **d**); *J*-EQE curves (**b**, **e**); Normalized EL spectra (**c**, **f**). In Supplementary Fig. 8d, a representative *J-V* curve of 5AVA-device is presented for comparison. These results indicate that loading the same amounts of BA or PA as 5AVA has negative effects on EQE values radiance.


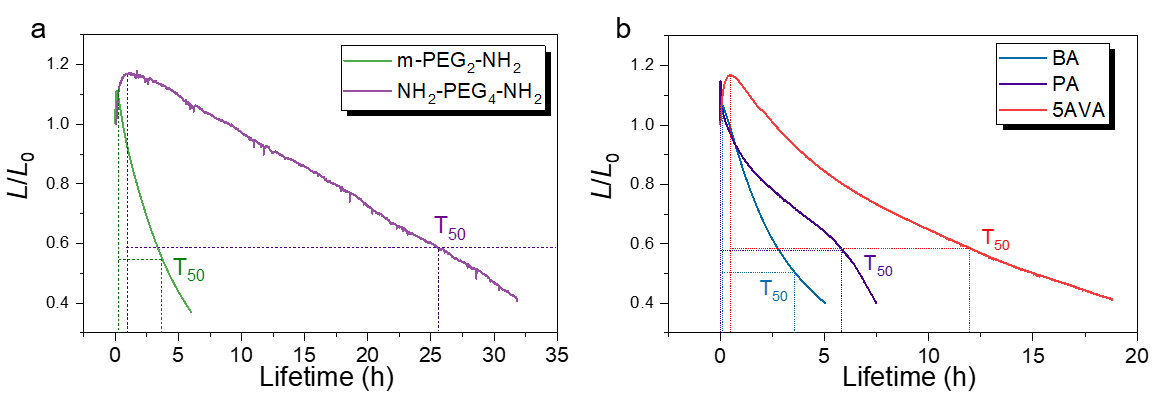


**Supplementary Figure 9 Operational lifetime of the representative devices fabricated with CAs and their MFAs counterparts**. **a,** m-PEG_2_-NH_2_ and NH_2_-PEG_4_-NH_2_. **b,** BA, PA, and 5AVA. The operational lifetime was measured in a N_2_-filled glove-box (O_2_ < 0.1 ppm, H_2_O < 0.1 ppm) at a constant current density of 20 mA cm^-2^. Here, T_50_ is defined as the time required to decay to half of its highest brightness.


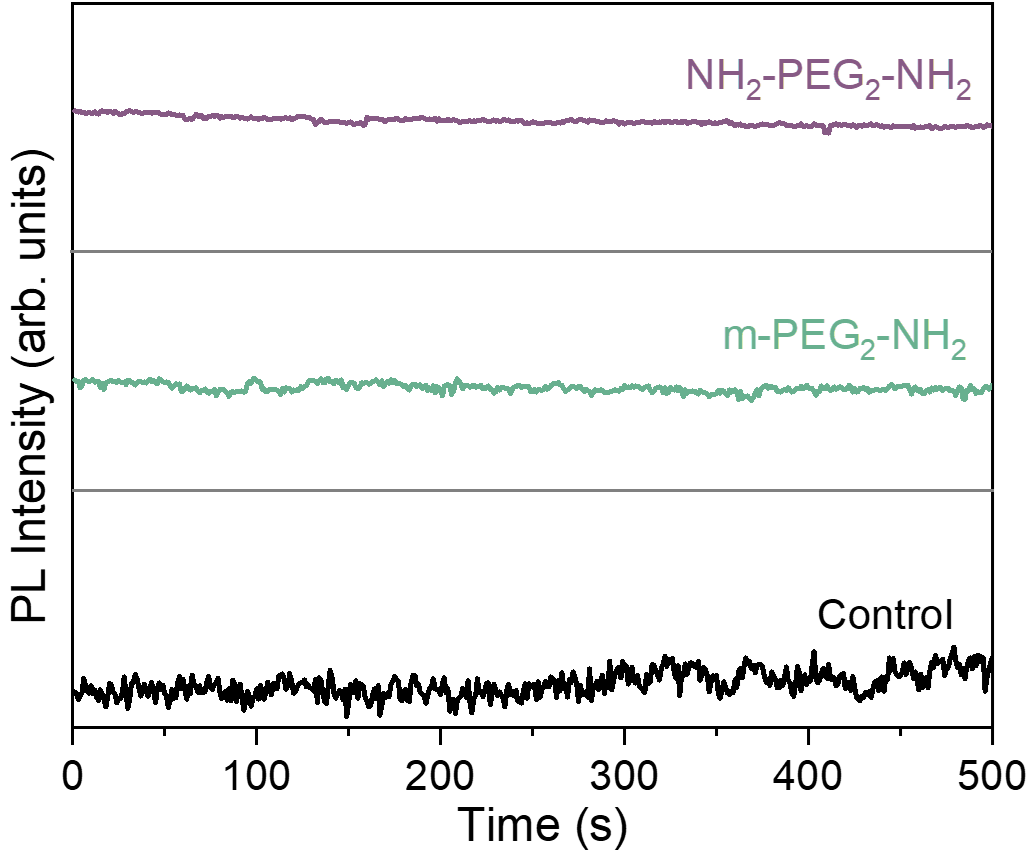


**Supplementary Figure 10 PL intensity evolution of the perovskite films (control, m-PEG_2_-NH_2,_ and NH_2_-PEG_4_-NH_2_) with time.** The data were recorded from PL mapping tests. All the perovskite films were deposited on ITO/ZnO/PEIE substrates and measured in ambient with encapsulation by epoxy and glass slides.


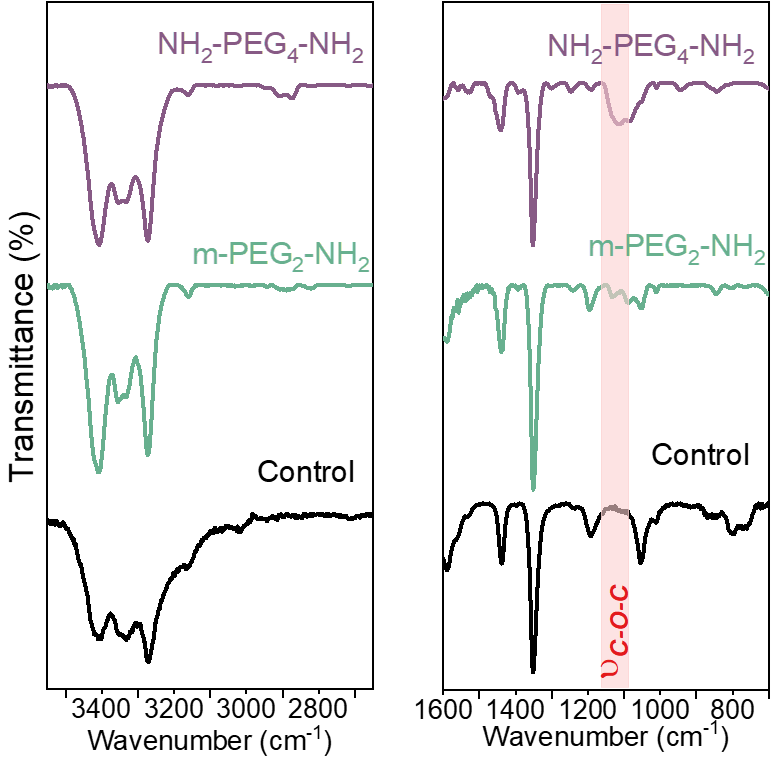


**Supplementary Figure 11 FT-IR spectra of control, m-PEG_2_-NH_2_-_,_ and NH_2_-PEG_4_-NH**_2_**-based perovskite films.** The pink square highlights the stretching vibration of ether (C-O-C) bonds. All the perovskite films were prepared from the respective optimal precursor stoichiometry as summarized in Supplementary Table 1.


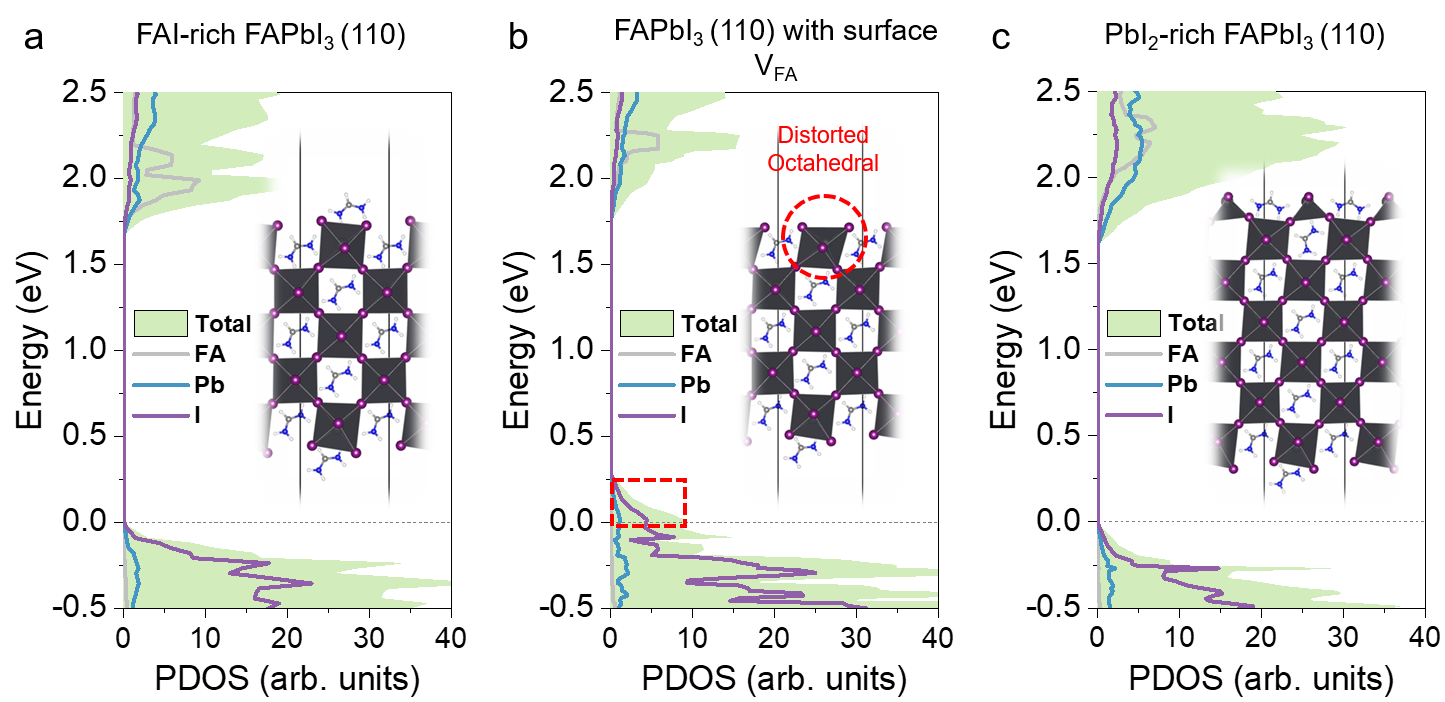


**Supplementary Figure 12 Projected density of states (PDOS) and optimized crystal structures (inner) for FAPbI_3_ (110) slab with different surface terminations. a**, FAI-rich surface. **b**, With surface FA^+^ vacancies (V_FA_). **c**, Pb-rich surface. The valence band maximum (VBM) and the conduction band minimum (CBM) of the cubic-FAPbI_3_ are mainly composed of I-5*p*, Pb-6*s,* and Pb-6*p* orbitals. Here, the presence of surface FA^+^ vacancies (V_FA_) leads to lattice distortion (highlighted in a red circle) and thus brings about hole trapping states (highlighted in a red rectangle).


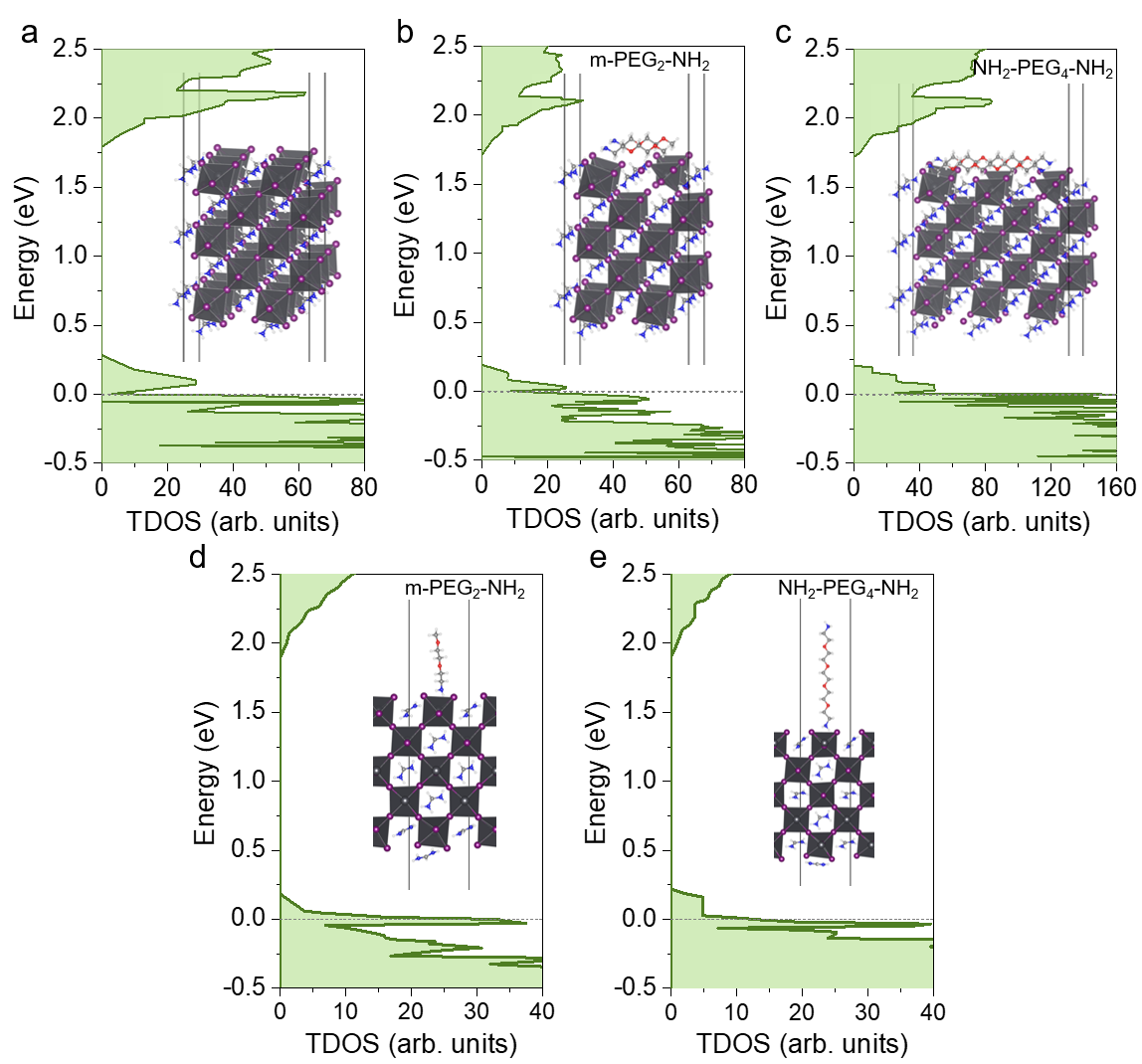


**Supplementary Figure 13 PDOS for V_FA_-terminated (110) FAPbI_3_ slabs with and without molecule adsorption. a,** A clean surface with V_FA_. **b, c,** With molecule adsorption paralleled to the surface: two m-PEG_2_-NH_2_ molecules (**b**); two NH_2_-PEG_4_-NH_2_ molecules (**c**). **d**, **e**, With molecule adsorption perpendicular to the surface: a m-PEG_2_-NH_2_ molecule (**d**); a NH_2_-PEG_4_-NH_2_ molecule (**e**).


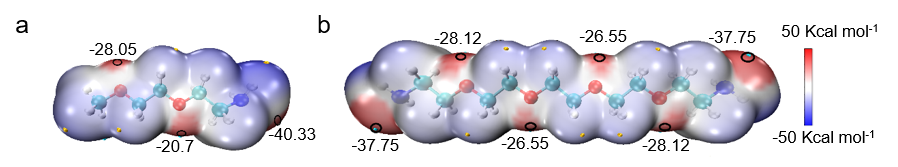


**Supplementary Figure 14 Simulated electrostatic potential surfaces of O and N atoms in additives. a,** m-PEG_2_-NH_2_. **b**, NH_2_-PEG_4_-NH_2_.

**Supplementary Figure 15 Digital photos of various solutions.** **a**, PbI_2_ solution. **b,** PbI_2_: NH_2_-PEG_4_-NH_2_ mixed solution. **c**, PbI_2_: NH_2_-PEG_4_-NH_2_: FAI mixed solution.


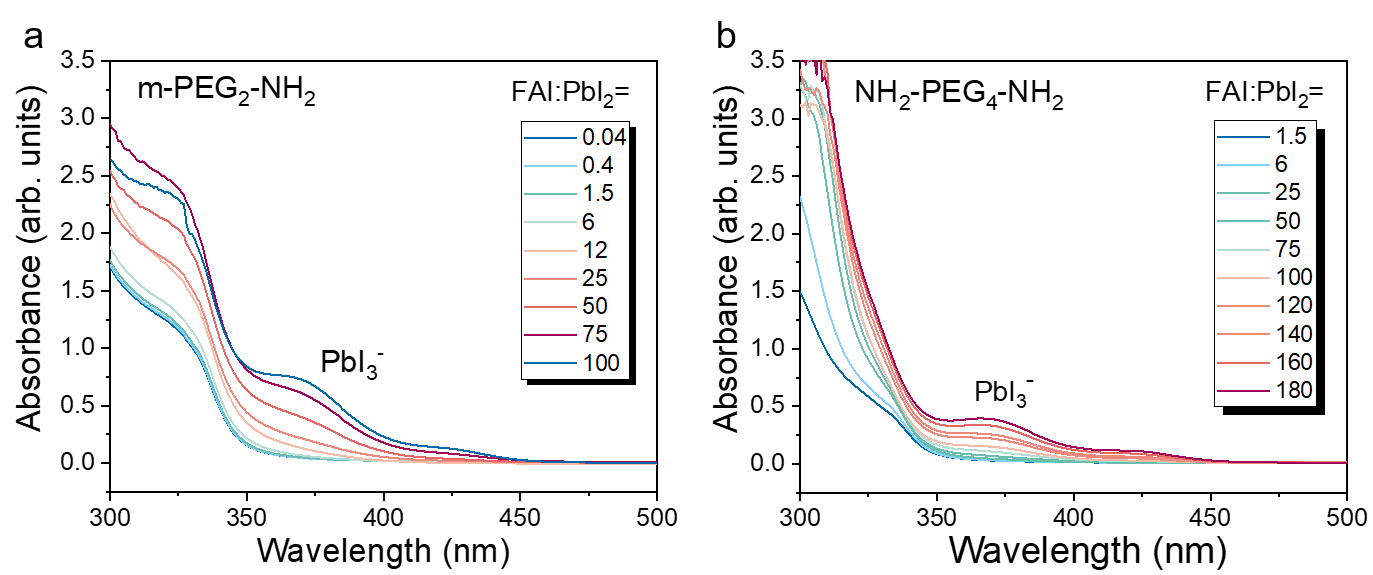


**Supplementary Figure 16 UV-vis absorbance traces for the different parental solutions with increasing [FAI]: [PbI_2_]**. **a**, PbI_2_: m-PEG_2_-NH_2_ (1: 10 in mole). **b**, PbI_2_: NH_2_-PEG_4_-NH_2_ (1: 5 in mole). The concentrations of PbI_2_ in both cases are 0.25 mM.


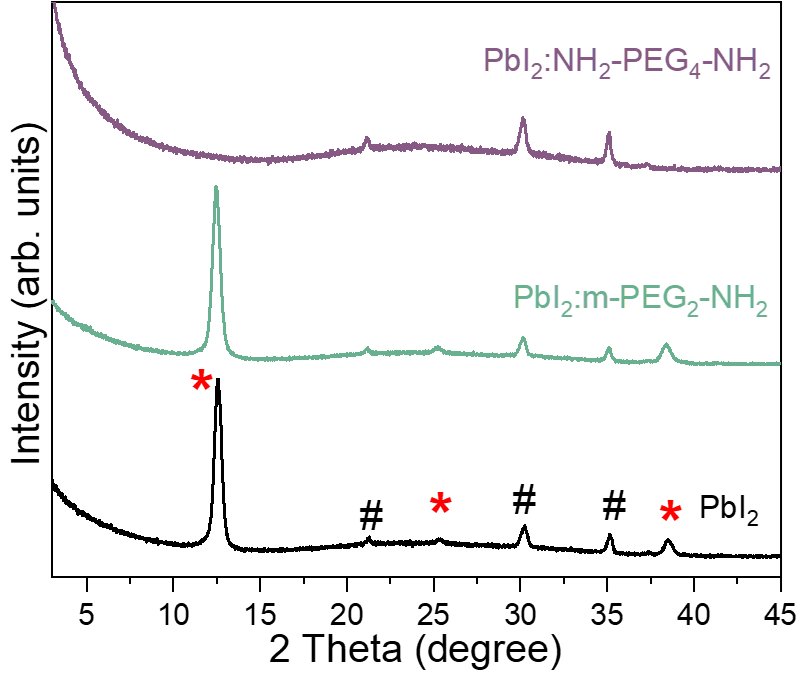


**Supplementary Figure 17 XRD patterns of neat PbI_2_ film, PbI_2_: m-PEG_2_-NH_2_ (1: 0.4) and PbI_2_: NH_2_-PEG_4_-NH_2_ (1: 0.2) films.** Here, # donates the diffraction peaks from ITO/ZnO/PEIE substrate and * donates the diffraction peaks from PbI_2_.


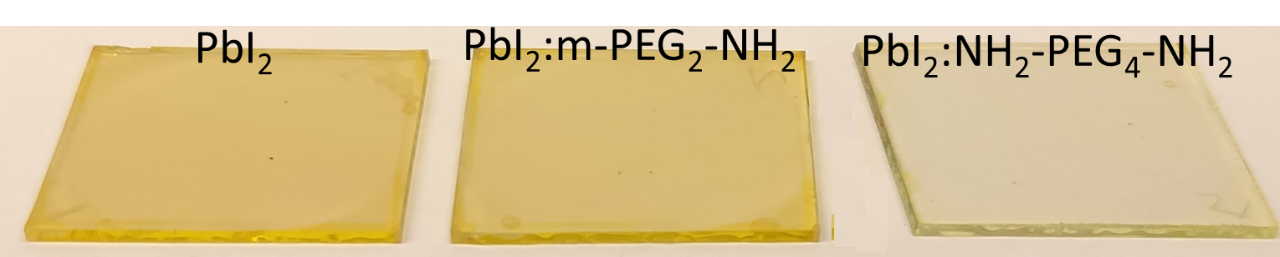


**Supplementary Figure 18 Digital images for PbI_2_ film, PbI_2_: m-PEG_2_-NH_2_ (1: 0.4 in mole) film, and PbI_2_: NH_2_-PEG_4_-NH_2_ (1: 0.2 in mole) films.** All the samples were deposited on ITO/ZnO/PEIE substrates and annealed at 100 ^o^C for 10 min before measurement.


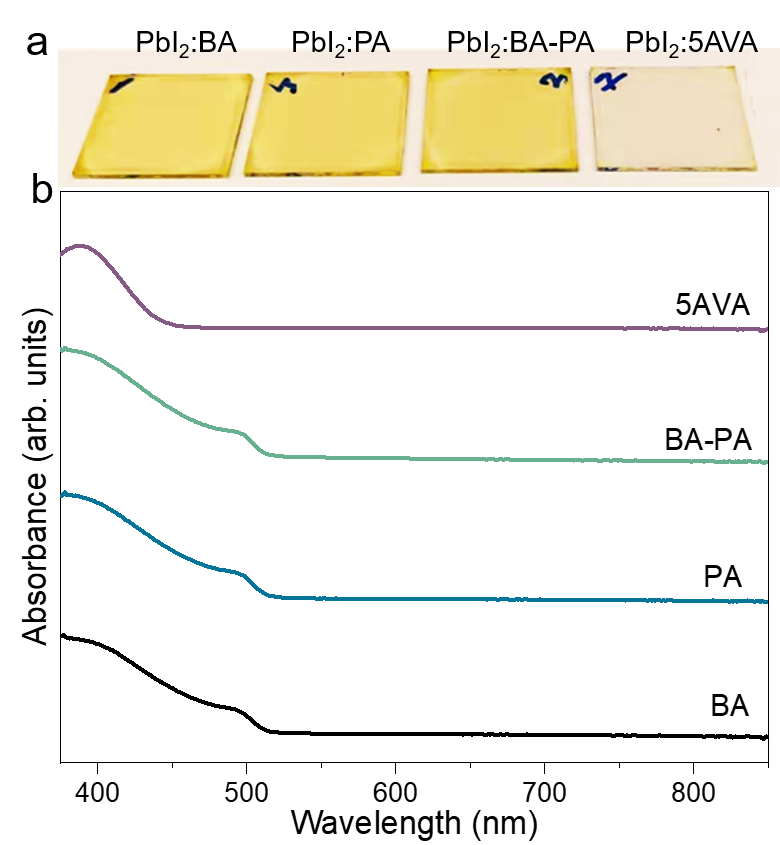


**Supplementary Figure 19 Characterizations of various PbI_2_: additive films. a,** Digital images of the films of interest. From left to right are PbI_2_: BA, PbI_2_: PA, PbI_2_: BA-PA, and PbI_2_: 5AVA, respectively. The feed ratios of additives are identical to those for device fabrication (See Supplementary Table 1). **b,** The corresponding UV-vis absorption spectra of PbI_2_: additive films. All the samples were deposited on ITO/ZnO/PEIE substrates and annealed at 100 ^o^C for 10 min before measurement.


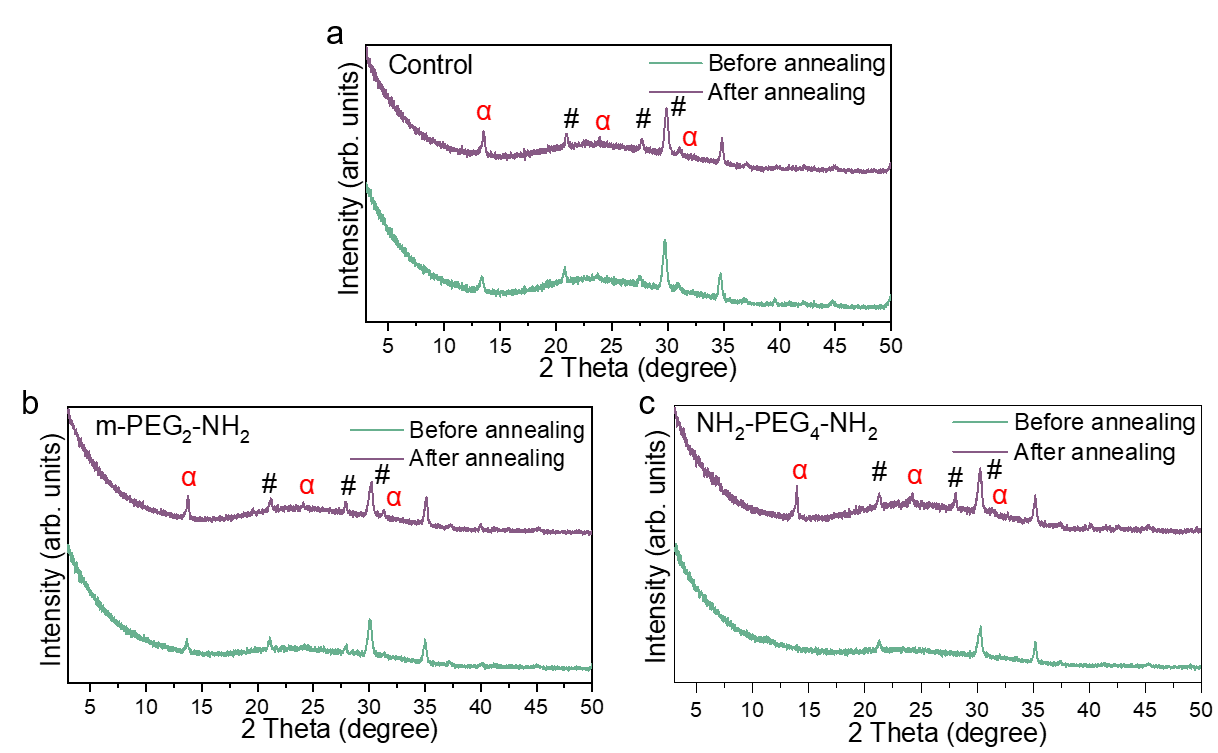


**Supplementary Figure 20 XRD patterns of perovskite films with additives before and after thermal annealing.** **a**, Control. **b**, m-PEG_2_-NH_2_. **c**, NH_2_-PEG_4_-NH_2_. All the samples were deposited on ITO/ZnO/PEIE substrates. Here, # donates the diffraction from ITO/ZnO/PEIE substrates, α donates the diffraction peaks from α-phase of FAPbI_3_.


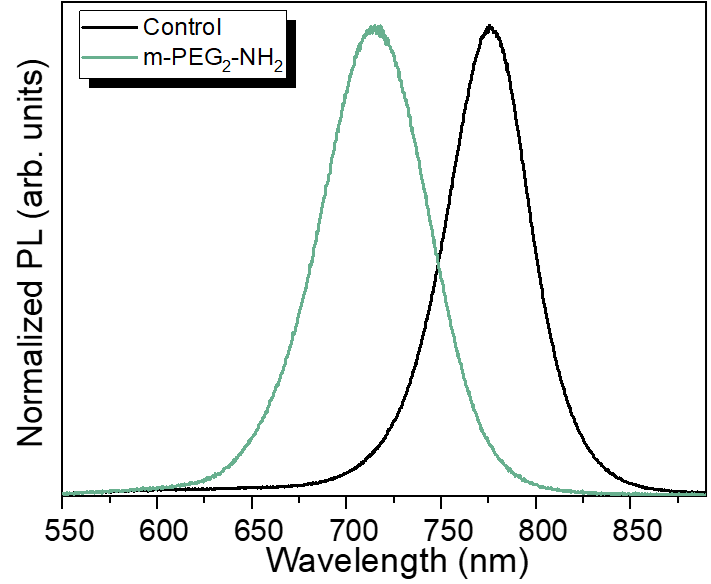


**Supplementary Figure 21 PL spectra of control and PbI_2_: FAI: m-PEG_2_-NH_2_ based precursor films without annealing.** All the perovskite films were deposited on ITO/ZnO/PEIE substrates.

**Supplementary Figure 22 Digital photos for precursor solutions (control, m-PEG_2_-NH_2_, NH_2_-PEG_4_-NH_2_) excited by a 635 nm laser.** Here, as no “Tyndall effect” is visible in the solution, the nano-particles indicated by TEM measurements (Figs. 3b and 3c in the main text) form during the spin-coating process.

**Supplementary Figure 23** **UV-vis absorption spectrum of PbI_2_: FAI: m-PEG_2_-NH_2_ (1: 2: 0.6 in mole) precursor film without annealing.** The film was prepared on ITO/ZnO/PEIE substrate. The concentration of PbI_2_ is 0.12 M.


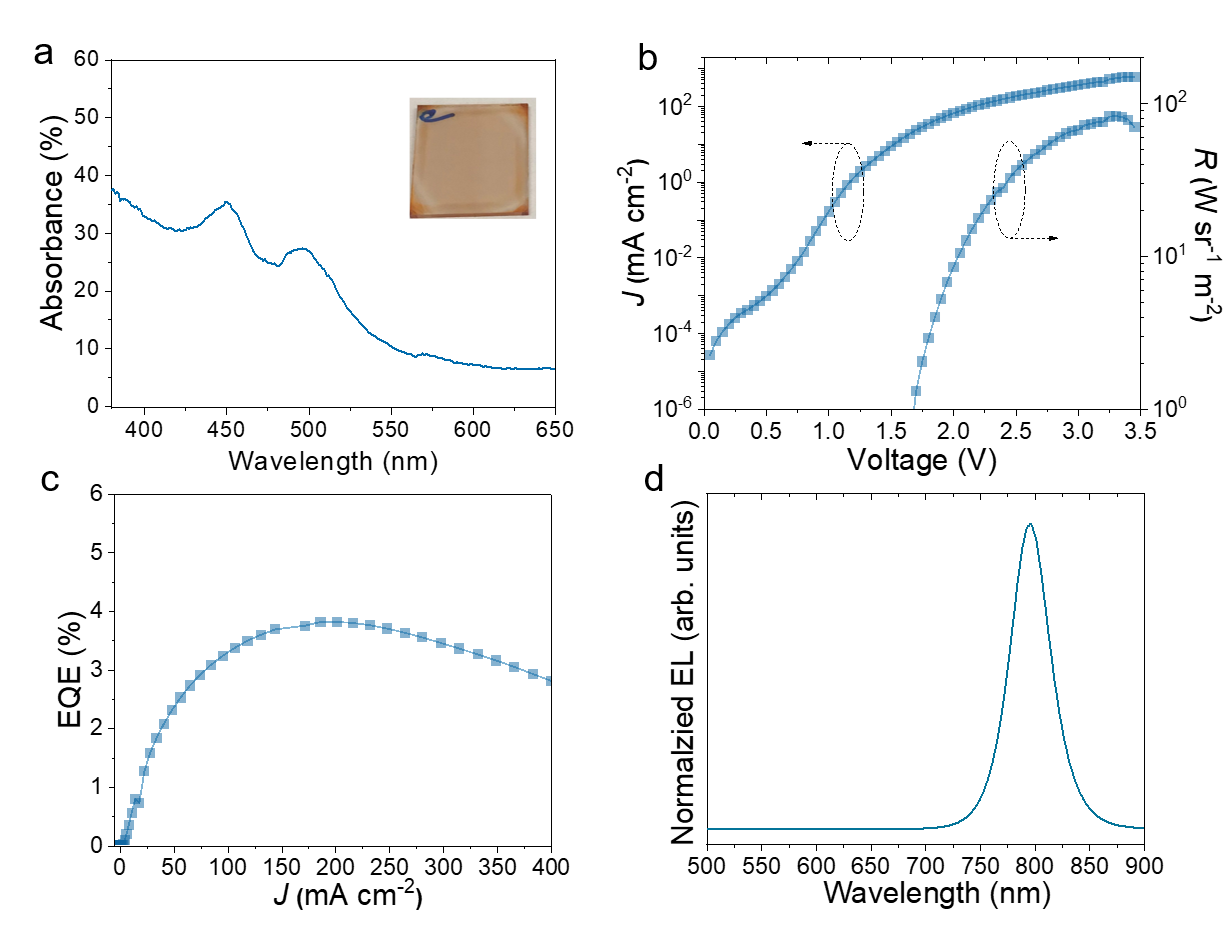


**Supplementary Figure 24** **The effects of DMSO addition. a.** UV-vis absorption spectra of PbI_2_: FAI: DMSO (1: 2: 1 in mole) precursor film without annealing. The inset shows a digital photo of the precursor film. **b-d**, Performance of representative DMSO based PeLEDs: *J-V-R* curves (**b**); *J*-EQE curve (**c**); Normalized EL spectra (**d**).


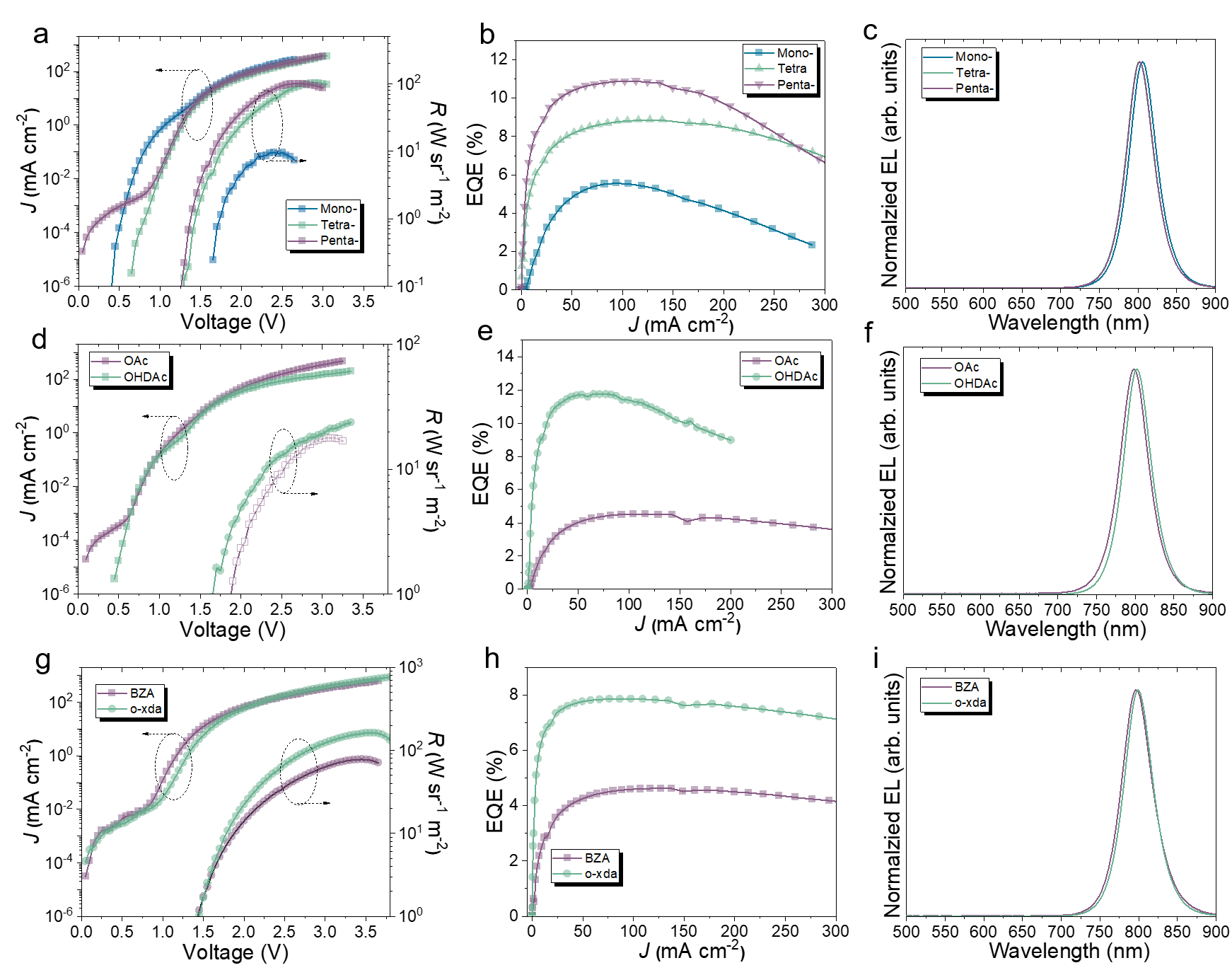


**Supplementary Figure 25 Performance of representative PeLEDs fabricated with various additives**. **a-c,** acrylate-based PeLEDs. **d-f,** OAc- and OHDAc-based PeLEDs. **g-i,** BZA- and o-xda-based PeLEDs. Device characteristics: *J-V-R* curves (**a**, **d**, **g**); *J*-EQE curves (**b**, **e**, **h**); Normalized EL spectra (**c**, **f**, **i**).


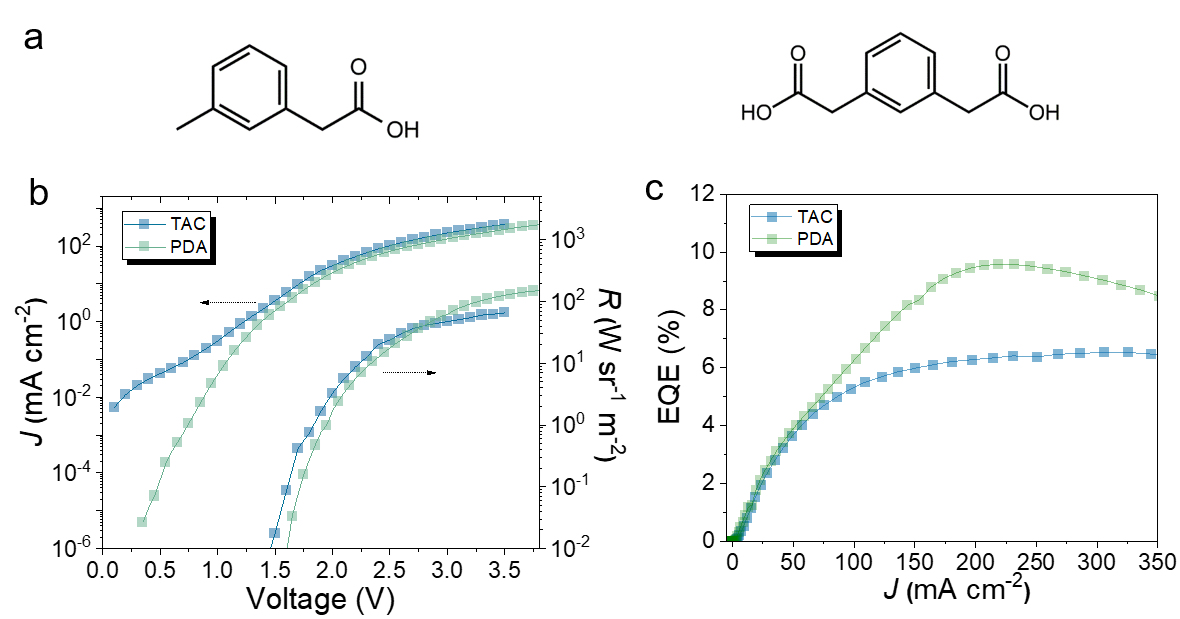


**Supplementary Figure 26 Characteristics of representative devices with aromatic acids**. **a**, Molecular structure of m-tolylacetic acid (TAC) and 1,3-phenylenediacetic acid (PDA). **b**, *J-V-R* curves, c, *J*-EQE curves.

**
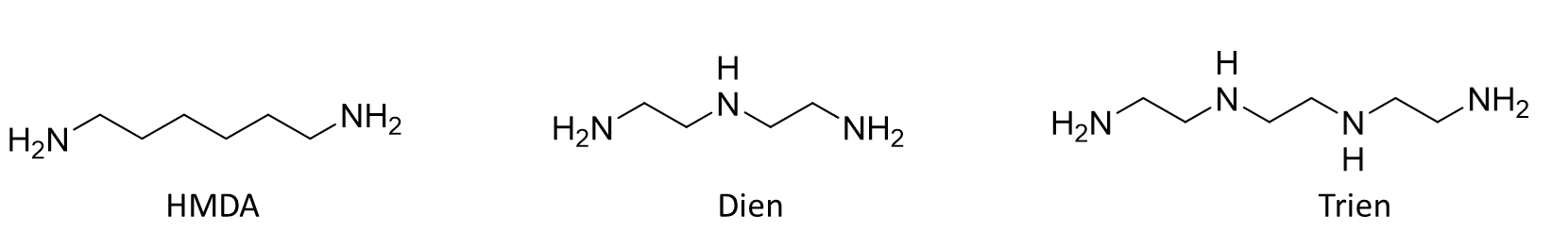

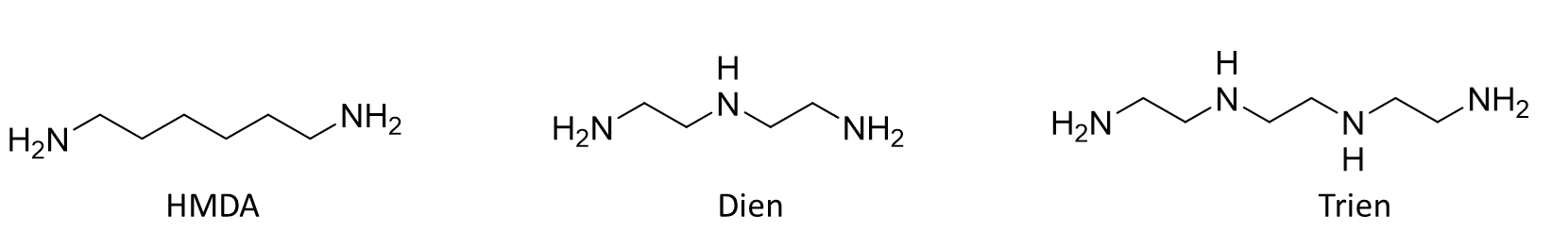
**

**Supplementary Figure 27 Molecular structures of the multidentate chelating molecules of HMDA and Trien.**

**Supplementary Figure 28 Digital photo of perovskite precursor (PbI_2_: FAI) solutions before and after Trien addition.**

**Supplementary Figure 29 UV-vis absorption spectrum of PbI_2_: FAI: HMDA (1: 2: 0.2) films before annealing.** The concentration of PbI_2_ is 0.12 M.


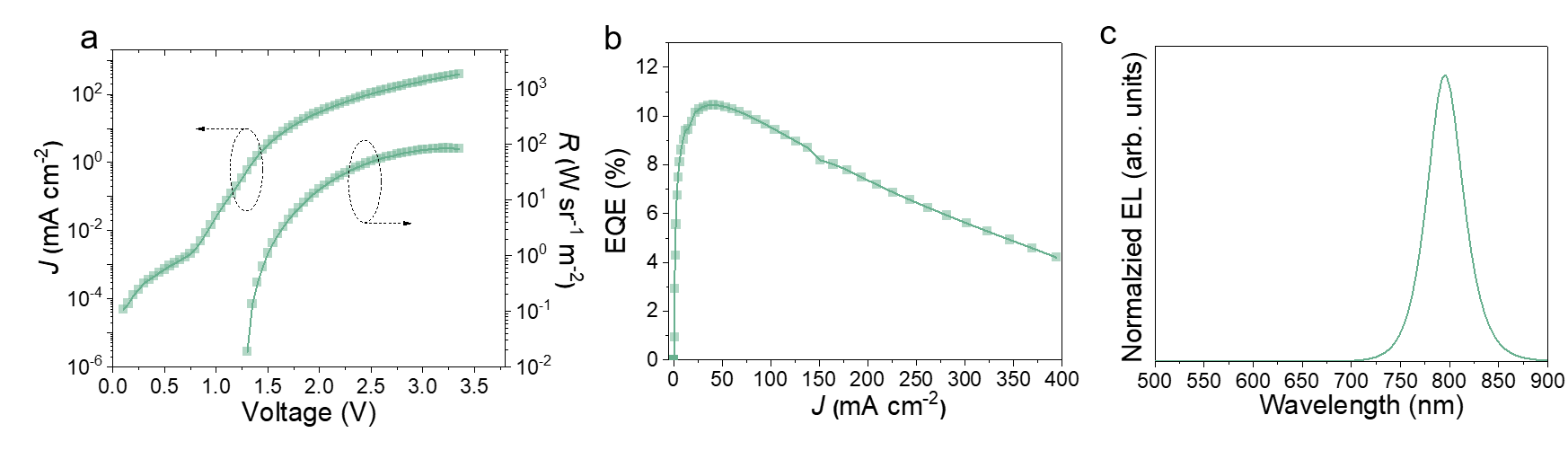


**Supplementary Figure 30 Performance of the representative HMDA based PeLED**. **a**, *J-V-R* curves. **b**, *J*-EQE curve. **c**, Normalized EL spectra.

**Supplementary Tables**

**Supplementary Table 1** A summary of optimized recipes for perovskite precursors with various additives. Notably, the determination of the optimized recipe considers both the peak EQE values and radiance.

| Precursors | FAI (mmol) | PbI_2_ (mmol) | Additives (mmol) |
| --- | --- | --- | --- |
| Control | 0.24 | 0.12 | N/O |
| BA | 0.24 | 0.12 | 0.048 |
| PA | 0.24 | 0.12 | 0.012 |
| m-PEG_2_-NH_2_ | 0.24 | 0.12 | 0.024 |
| m-PEG_4_-NH_2_ | 0.24 | 0.12 | 0.012 |
| 5AVA | 0.24 | 0.12 | 0.072 |
| NH_2_-PEG_4_-NH_2_ | 0.24 | 0.12 | 0.024 |
| DMSO | 0.24 | 0.12 | 0.120 |
| Mono-acrylate | 0.24 | 0.12 | 0.096 |
| Tetra-acrylate | 0.24 | 0.12 | 0.024 |
| Penta-acrylate | 0.24 | 0.12 | 0.018 |
| OAc | 0.24 | 0.12 | 0.012 |
| OHDAc | 0.24 | 0.12 | 0.009 |
| BZA | 0.24 | 0.12 | 0.036 |
| o-xda | 0.24 | 0.12 | 0.024 |
| HMDA | 0.24 | 0.12 | 0.024 |

**Supplementary Table 2** A summary of PeLED characteristics with different additives.

| Additives | | EL (nm) | FWHM (nm) | | R (W sr^-1^ m^-2^) | | EQE (%) |
| --- | --- | --- | --- | --- | --- | --- | --- |
| Control^a^ | | 802 | 42 | 80 ± 40 (144) | | 5.3 ± 0.7 (7.0) | |
| BA^a^ | | 800 | 42 | 156 ± 28 (190) | | 7.1 ± 0.7 (8.1) | |
| PA^a^ | | 802 | 43 | 135 ± 24 (165) | | 6.5 ± 0.6 (7.4) | |
| BA+PA^a^ | | 802 | 43 | 108 ± 32 (158) | | 7.6 ± 0.5 (8.3) | |
| 5AVA^a^ | | 795 | 43 | 173 ± 20 (204) | | 15.5 ± 0.8 (17.2) | |
| m-PEG_2_-NH_2_^a^ | | 802 | 42 | 80 ± 25 (108) | | 11.0 ± 0.5 (11.7) | |
| m-PEG_4_-NH_2_^a^ | | 802 | 42 | 78 ± 20 (102) | | 11.2 ± 0.7 (12.0) | |
| NH_2_-PEG_4_-NH_2_^a^ | | 802 | 42 | 360 ± 27 (393) | | 19.2 ± 0.6 (20.2) | |
| DMSO^b^ | | 796 | 43 | 72 ± 14 (82) | | 4.2 ± 0.3 (4.6) | |
| Mono-acrylate^b^ | | 805 | 42 | 42 ± 8 (52) | | 4.5 ± 0.4 (5.8) | |
| Tetra-acrylate^b^ | | 802 | 42 | 94 ± 23 (136) | | 9.9 ± 0.4 (10.5) | |
| Penta-acrylate^b^ | | 802 | 42 | 94 ± 3 (96) | | 10.5 ± 0.4 (11.1) | |
| OA^b^ | | 799 | 44 | 45 ± 5 (52) | | 4.1 ± 0.3 (4.6) | |
| OHDAC^b^ | | 802 | 42 | 123 ± 5 (130) | | 11.1 ± 0.5 (11.7) | |
| TAC^b^ | | 801 | 43 | 50 ± 12 (65) | | 5.1 ± 0.5 (6.5) | |
| PDA^b^ | 802 | | 42 | 178 ± 10 (191) | | 8.9 ± 0.6 (9.6) | |
| BZA^b^ | 797 | | 47 | 78 ± 6 (84) | | 4.2 ± 0.3 (4.7) | |
| O-XDA^b^ | 800 | | 44 | 152 ± 8 (162) | | 7.3 ± 0.4 (7.9) | |
| HMDA^b^ | 796 | | 43 | 85 ± 8 (92) | | 10.8 ± 0.5 (11.4) | |

^a^ Summarized from twenty individual devices; ^b^ Summarized from six individual devices.

**Supplementary Table 3** Calculated binding energy of m-PEG_2_-NH_2_ and NH_2_-PEG_4_-NH_2_ on (110) adsorbed on FAPbI_3_ (110) slab.

| Additives | Binding energy  (parallel to the surface, eV) | Binding energy  (perpendicular to the surface, eV) |
| --- | --- | --- |
| m-PEG_2_-NH_2_ | 0.87 | 0.57 |
| NH_2_-PEG_4_-NH_2_ | 1.13 | 0.52 |

**Supplementary Notes**

**Supplementary Note 1. The role of O atoms and characterizations of PeLEDs prepared with m-PEG_4_-NH_2_ as the additive**

Oxygen atoms are electron-rich moieties and thus may have an impact on crystallization dynamics as well. To provide a rationale for the effects of O atoms, we explore another amine counterpart with longer polyethylene glycol (PEG) chain compared to m-PEG_2_-NH_2_, that is, 2,5,8,11-tetraoxatridecan-13-amine (m-PEG_4_-NH_2_). Despite the increase in the coordination number, m-PEG_4_-NH_2_ addition gives no noticeable difference compared to its counterpart in terms of film morphology, crystal quality, and device performance (Supplementary Figs 1-3, and Supplementary Fig 7). The visible excitonic features in the absorption spectrum of precursor films also confirm a week interaction between lead cations and m-PEG_4_-NH_2_, analogs to m-PEG_2_-NH_2_. As such, we conclude that the effects of oxygen atoms on perovskite crystallization are not as significant as amino groups in the current case. For simplicity, we regard m-PEG_2_-NH_2_ and m-PEG_4_-NH_2_ as mono-functional additives here.

**Supplementary Note 2. The impact of perovskite film morphology on device performance.**

Since the chelating additives lead to smaller crystal sizes and better surface coverage than the MFA-based counterparts, one may concern that the high device performance of CA-devices is mainly due to the differences in film morphology. By using the anti-solvent treatment with chlorobenzene, we observe reduced crystal size and dramatically improved coverage of the resultant perovskite films from the same precursor solutions (Supplementary Fig. 3 and Supplementary Figs. 4a and b). However, the EQE values of devices with anti-solvent treatment do not exhibit much difference (Supplementary Figs. 4e-f). Therefore, we believe that the morphology variations in Supplementary Fig. 3 are not the main reason for such a big difference in the performance between CAs- and MFAs-based devices.

**Supplementary Note 3. Analysis of ultraviolet photoelectron spectroscopy** (**UPS) results**

We UPS to investigate the effect of additive addition on the electronic properties of perovskite films. An optimized feed ratio of PbI_2_: FAI: additive = 1: 2: 0.2 is employed for both m-PEG_2_-NH_2_ and NH_2_-PEG_4_-NH_2_ cases. The UPS spectra and the extracted energy levels with respect to Femi level (E_F_) have been presented in Supplementary Fig. 5c and 5d, respectively. The control films show a work function (WF) of 4.00 eV and a VB leading edge of -1.05 eV below E_F_, which corresponds to ionization energy (IE) of 5.05 eV. Notably, the additive addition leads to the shifts of vacuum level, as indicated by the changes in WFs. In addition, the VB edge slightly downshifts to -1.09 eV and -1.16 eV for m-PEG_2_-NH_2_ and NH_2_-PEG_4_-NH_2_ cases, respectively. Accordingly, the IE value is 4.90 eV for m-PEG_2_-NH_2_ films and 4.86 eV for NH_2_-PEG_4_-NH_2_ ones. These discrepancies can be assigned to the formation of interfacial dipole due to the introduction of polar functional groups from the additives, an *n*-doping effect as a result of the changes in defect sites (*e.g.* the density and the type of defects) or a collective effect of them^2-4^. The small differences in the electronic states of additive-based perovskite films are hardly possible to be responsible for the significant difference in device performance.

**Supplementary Note 4. Analysis of DFT calculations**

In order to assess the contribution of passivation effects on reducing non-radiative recombination of the perovskites, we performed density functional theory (DFT) calculations^5^. In Supplementary Fig. 12, we show the projected density of states (PDOS) for FAPbI_3_ (110) slab with different surface terminations, including FAI-rich surface, FA vacancies (V_FA_)-terminated surface, and Pb-rich surface. We find that the surface V_FA_ can generate hole trapping states above the VBM as a result of distorted octahedral, while the others do not create trap states within the bandgap. We thus proceeded to investigate the changes of electronic states of V_FA_-terminated structure with different molecule adsorption. Two types of molecular adsorption configurations are considered, *i.e.*, in parallel and perpendicular to the surface (Supplementary Fig. 13). A clean surface without molecule adsorption is investigated as well. The adsorption binding energy is summarized in Supplementary Table 3. Notably, this calculated value is to quantify the adsorption energy of molecules on the perovskite surface, which is different from the binding energy between these molecules and lead cations in the precursors. Compared to the clean surface, which shows a high density of trap states, the molecular adsorption effectively weakens the hole trapping due to the suppression of lattice distortion. Nevertheless, no significant difference in the energy depth and density of trap states can be observed between these two molecules regardless of the adsorption type. We thus speculate that the different passivation effectiveness between m-PEG_2_-NH_2_ and NH_2_-PEG_4_-NH_2_ may not be the major reason for the huge discrepancies in device performance. Given that the functional moieties in these two molecules share a very similar chemical environment, as shown in Supplementary Fig. 14, the small difference in passivation effectiveness is reasonable.

**Supplementary References**

1. Xu, W. *et al.* Rational molecular passivation for high-performance perovskite light-emitting diodes. *Nat. Photon.* **13**, 418-424 (2019).
2. Zhou, Y. *et al.* A universal method to produce low–work function electrodes for organic electronics. *Science* **336**, 327 (2012).
3. Wang, Q. *et al.* Qualifying composition dependent p and n self-doping in CH_3_NH_3_PbI_3_. *Appl. Phys. Lett.* **105**, 163508 (2014).
4. Hawash, Z. *et al.* Interfacial modification of perovskite solar cells using an ultrathin mai layer leads to enhanced energy level alignment, efficiencies, and reproducibility. *J. Phys. Chem. Lett.* **8**, 3947-3953 (2017).
5. Kim, J., Lee, S.-C., Lee, S.-H. & Hong, K.-H. Importance of orbital interactions in determining electronic band structures of organo-lead iodide. *J. Phys. Chem. C* **119**, 4627-4634 (2015).
