# Supplementary material for: Manipulating crystallization dynamics through chelating molecules for bright perovskite emitters
Source: Nat Commun. 2021 Aug 10;12:4831. doi: 10.1038/s41467-021-25092-7 (PMC8355273; doi:10.1038/s41467-021-25092-7)
Supplement: Supplementary file 2 — Description of Additional Supplementary Files [file 41467_2021_25092_MOESM2_ESM.pdf]

Description of Additional Supplementary Files

File Name: Supplementary Movie 1
Description: PL mapping for control films

File Name: Supplementary Movie 2
Description: PL mapping for m-PEG_2-NH_2 perovskite films

File Name: Supplementary Movie 3
Description: PL mapping for NH_2-PEG_4-NH_2 perovskite films
